# Formononetin relieves the facilitating effect of lncRNA AFAP1-AS1-miR-195/miR-545 axis on progression and chemo-resistance of triple-negative breast cancer

**DOI:** 10.18632/aging.203156

**Published:** 2021-07-21

**Authors:** Jingjing Wu, Wen Xu, Lina Ma, Jiayu Sheng, Meina Ye, Hao Chen, Yuzhu Zhang, Bing Wang, Mingjuan Liao, Tian Meng, Yue Zhou, Hongfeng Chen

**Affiliations:** 1 Department of Breast, Longhua Hospital Affiliated to Shanghai University of TCM, Shanghai, China; 2 State Key Laboratory of Bioreactor Engineering and Shanghai Key Laboratory of New Drug Design, School of Pharmacy, East China University of Science and Technology, Shanghai, China; 3 Department of Breast Surgery, Shanghai Yueyang Hospital of Integrated Traditional Chinese and Western Medicine, Shanghai, China; 4 Department of Mammary Disease, Guangdong Provincial Hospital of Chinese Medicine, Guangzhou, Guangdong, China; 5 Department of Traditional Chinese Medicine, The Ninth People's Hospital, Medical School of Shanghai Jiaotong University, Shanghai, China

**Keywords:** lncRNA AFAP1-AS1, miR-195/miR-545, triple-negative breast cancer, formononetin, chemo-resistance

## Abstract

This investigation attempted to discern whether formononetin restrained progression of triple-negative breast cancer (TNBC) by blocking lncRNA AFAP1-AS1-miR-195/miR-545 axis. We prepared TNBC cell lines (i.e. MDA-MB-231 and BT-549) and normal human mammary epithelial cell line (i.e. MCF-10A) in advance, and the TNBC cell lines were, respectively, transfected by pcDNA3.1-lncRNA AFAP1-AS1, si-lncRNA AFAP1-AS1, pcDNA6.2/GW/EmGFP-miR-545 or pcDNA6.2/GW/EmGFP-miR-195. Resistance of TNBC cells in response to 5-Fu, adriamycin, paclitaxel and cisplatin was evaluated through MTT assay, while potentials of TNBC cells in proliferation, migration and invasion were assessed via CCK8 assay and Transwell assay. Consequently, silencing of lncRNA AFAP1-AS1 impaired chemo-resistance, proliferation, migration and invasion of TNBC cells (*P*<0.05), and over-expression of miR-195 and miR-545, which were sponged and down-regulated by lncRNA AFAP1-AS1 (*P*<0.05), significantly reversed the promoting effect of pcDNA3.1-lncRNA AFAP1-AS1 on proliferation, migration, invasion and chemo-resistance of TNBC cells (*P*<0.05). Furthermore, CDK4 and Raf-1, essential biomarkers of TNBC progression, were, respectively, subjected to target and down-regulation of miR-545 and miR-195 (*P*<0.05), and they were promoted by pcDNA3.1-lncRNA AFAP1-AS1 at protein and mRNA levels (*P*<0.05). Additionally, formononetin significantly decreased expressions of lncRNA AFAP1-AS1, CDK4 and Raf-1, while raised miR-195 and miR-545 expressions in TNBC cells (*P*<0.05), and exposure to it dramatically contained malignant behaviors of TNBC cells (*P*<0.05). In conclusion, formononetin alleviated TNBC malignancy by suppressing lncRNA AFAP1-AS1-miR-195/miR-545 axis, suggesting that molecular targets combined with traditional Chinese medicine could yield significant clinical benefits in TNBC.

## INTRODUCTION

Triple-negative breast cancer (TNBC), responsible for around 15% of global breast cancer (BC) cases, is histopathologically featured by shortages of estrogen receptor (ER), progesterone receptor (PR) and human epidermal growth factor receptor (HER)-2 [1]. Given its insensitivity responding to HER2-targeted therapy and endocrine therapy, TNBC was principally tackled by chemotherapies founded upon anthracycline and taxane [2], whose clinical efficacy, nonetheless, turned less encouraging than desired owing to development of drug resistance [3, 4]. As a consequence, profound comprehension of drug-resistance is indispensable to perfect strategies for TNBC treatment.

It was documented that organisms at high evolutionary levels usually possessed a large proportion of non-coding (nc) RNAs in their genome [5]. For example, the ratio of ncRNAs in human genome, which was in excess of 70%, far surpassed 5% in nematode genome and 25% in zebrafish genome [6, 7], implying that ncRNAs were vital players in the pathophysiology of highly-evolved human beings. Long-chain non-coding RNAs (lncRNAs), implicated in carcinogenesis at transcriptional and post-transcriptional levels [8], have been massively reported to behave well in signifying BC onset and exacerbation [9–11]. For instance, up-regulation of lncRNA NF-kB interacting lncRNA (NKILA) was predictive of favorable survival among BC patients, and it undermined metastatic potential of BC cells through weakening transcriptional activity of NF-κB [12]. Moreover, forced expression of lncRNA actin filament-associated protein 1-antisense RNA1 (AFAP1-AS1), the antisense product of *AFAP1*, considerably deteriorated BC prognosis [13, 14] through mobilizing Wnt/β-catenin signaling [15], controlling miR-145/MTH1 axis [16], or promoting AUF1-mediated ERBB2 translation [17]. Beyond that, our previous microarray analysis (Supplementary Table 1) identified that lncRNA AFAP1-AS1 expression in cisplatin-resistant MDA-MB-231 (MDA-MB-231/DDP) cell line was around 8.22 folds of that in MDA-MB-231 cell line, hinting that lncRNA AFAP1-AS1 might empower drug-resistance in TNBC. However, detailed signaling networks controlled by lncRNA AFAP1-AS1 in manipulating TNBC chemo-resistance remained ambiguous.

In addition, the prominent role of traditional Chinese medicines (TCMs) in suppressing tumorigenesis has also been increasingly recognized at home and abroad. For instance, formononetin, an isoflavonoid isolated from astragalus membranaceus and spatholobus suberectus, was found to impair capabilities of BC cells in proliferating, migrating and invading via blockade of PI3K/Akt signaling [18, 19]. Synergy of formononetin with metformin or everolimus also pronouncedly antagonized growth of BC cells by depressing ERK1/2 signaling [20] and mTOR signaling [21]. Notably, exposure to formononetin could significantly alter miRNA profiling in human umbilical vein endothelial cells (HUVECs), such as elevating expressions of miR-375 and miR-200b [22], both of which were crucial protectors against BC progression [23, 24]. Despite these discoveries, lncRNAs, which were likely to act upon miRNAs through classical competing endogenous (ce) RNA manner [25], were barely explored regarding their implication in formononetin-involved BC inhibition, let alone lncRNA/miRNA axes.

To bridge this gap, this investigation was designed to unveil lncRNAs (e.g. lncRNA AFAP1-AS1) and associated miRNA networks that were involved in the protective impact of formononetin against TNBC development, which might be conducive to clinical treatment of TNBC.

## RESULTS

### Clinical implication of lncRNA AFAP1-AS1 in TNBC

LncRNA AFAP1-AS1 expression in TNBC tissues and non-TNBC tissues was significantly promoted as opposed to adjacent non-cancerous tissues (*P*<0.05), and lncRNA AFAP1-AS1 expression in TNBC tissues was around 3 folds of that in non-TNBC tissues (*P*<0.05) (Supplementary Figure 1A). According to Supplementary Table 2, TNBC patients were categorized into high-level (≥6.45) lncRNA AFAP1-AS1 group (n=51) and low-level (<6.45) lncRNA AFAP1-AS1 group (n=43), with mean lncRNA AFAP1-AS1 expression as the cut-off point. Analogously, the non-TNBC population was divided into high-level (≥1.78) lncRNA AFAP1-AS1 (n=78) group and low-level (<1.78) lncRNA AFAP1-AS1 (n=77) group, also utilizing their mean lncRNA AFAP1-AS1 expression as the demarcation point. It was indicated that high lncRNA AFAP1-AS1 level was associated with advanced histological grade (III vs. I+II: OR=3.37, 95%CI: 1.436-7.908), large tumor size (T3 vs. T1+T2: OR=2.462, 95%CI: 1.036-5.847), lymph-node metastasis (yes vs. no: OR=2.591, 95%CI: 1.126-5.963) and high proportion of Ki-67 (>14% vs. ≤14%: OR=2.516, 95% CI: 1.082-5.849) of TNBC patients in comparison to low lncRNA AFAP-AS1 level (all *P*<0.05), however, these associations were hardly discerned in the non-TNBC cohort (Supplementary Table 2). Moreover, Kaplan-Meier curve of TNBC population suggested that survival of patients in the low-level lncRNA AFAP1-AS1 group was prolonged when compared with patients of high-level lncRNA AFAP1-AS1 group (*P*<0.05) (Supplementary Figure 1B). The multivariate regression analyses further exposed that large tumor size (HR=1.785, 95%CI: 1.063-2.996), advanced clinical stage (HR=2.985, 95%CI: 1.772-5.028), lymph-node metastasis (HR=2.354, 95%CI: 1.408-3.933) and high lncRNA AFAP1-AS1 level (HR=2.6, 95%CI: 1.526-4.431) were independently symbolic of TNBC patients’ unfavorable 3-year survival in this Chinese cohort (Supplementary Table 3).

### Impact of lncRNA AFAP1-AS1 on chemo-sensitivity, proliferation, migration and invasion of TNBC cell lines

**Figure 1:**
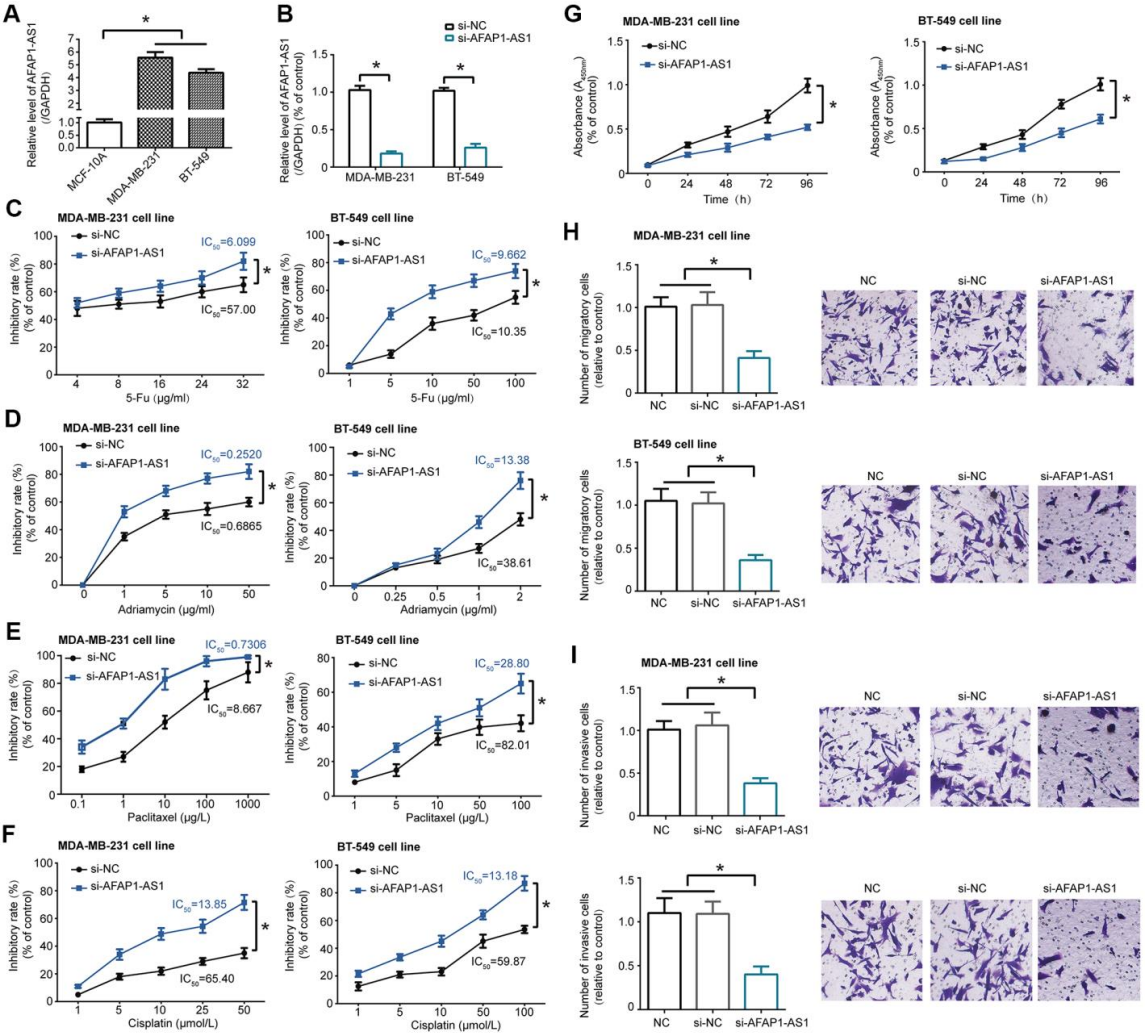
**LncRNA AFAP1-AS1 regulated chemo-sensitivity and activity of triple-negative breast cancer (TNBC) cell lines.** (**A**) LncRNA AFAP1-AS1 expression was up-regulated in TNBC cell lines (i.e. MDA-MB-231 and BT-549) as compared with normal breast epithelial cell line (i.e. MCF-10A). *: *P*<0.05. (**B**) LncRNA AFAP1-AS1 expression was decreased in MDA-MB-231 and BT-549 cell lines after transfection of si-lncRNA AFAP1-AS1. *: *P*<0.05. (**C**–**F**) Sensitivity of MDA-MB-231 and BT-549 cell lines responding to 5-Fu (**C**), adriamycin (**D**), paclitaxel (**E**) and cisplatin (**F**) was enhanced after transfection of si-lncRNA AFAP1-AS1. *: *P*<0.05. (**G**–**I**) Proliferation (**G**), migration (**H**) and invasion (**I**) of MDA-MB-231 and BT-549 cell lines were assessed after silencing of lncRNA AFAP1-AS1. *: *P*<0.05.

LncRNA AFAP1-AS1 expression in TNBC cell lines (i.e. MDA-MB-231 and BT-549) was obviously heightened as compared with normal breast epithelial cell line (i.e. MCF-10A) (*P*<0.05) (Figure 1A). Silencing of lncRNA AFAP1-AS1 (i.e. si-lncRNA AFAP1-AS1 group), which significantly decreased lncRNA AFAP1-AS1 expression in MDA-MB-231 and BT-549 cell lines (*P*<0.05) (Figure 1B), enhanced the toxic effect of 5-Fu (Figure 1C), adriamycin (Figure 1D), paclitaxel (Figure 1E) and cisplatin (Figure 1F) on MDA-MB-231 and BT-549 cell lines, leading to smaller IC50 values than si-NC group (all *P*<0.05). Furthermore, viability (Figure 1G), migration (Figure 1H) and invasion (Figure 1I) of MDA-MB-231 and BT-549 cell lines were notably suppressed after transfection of si-lncRNA AFAP1-AS1, when compared with si-NC group (all *P*<0.05).

### LncRNA AFAP1-AS1 sponged miR-545-3p/miR-195 and reduced their expression in TNBC cell lines

**Figure 2:**
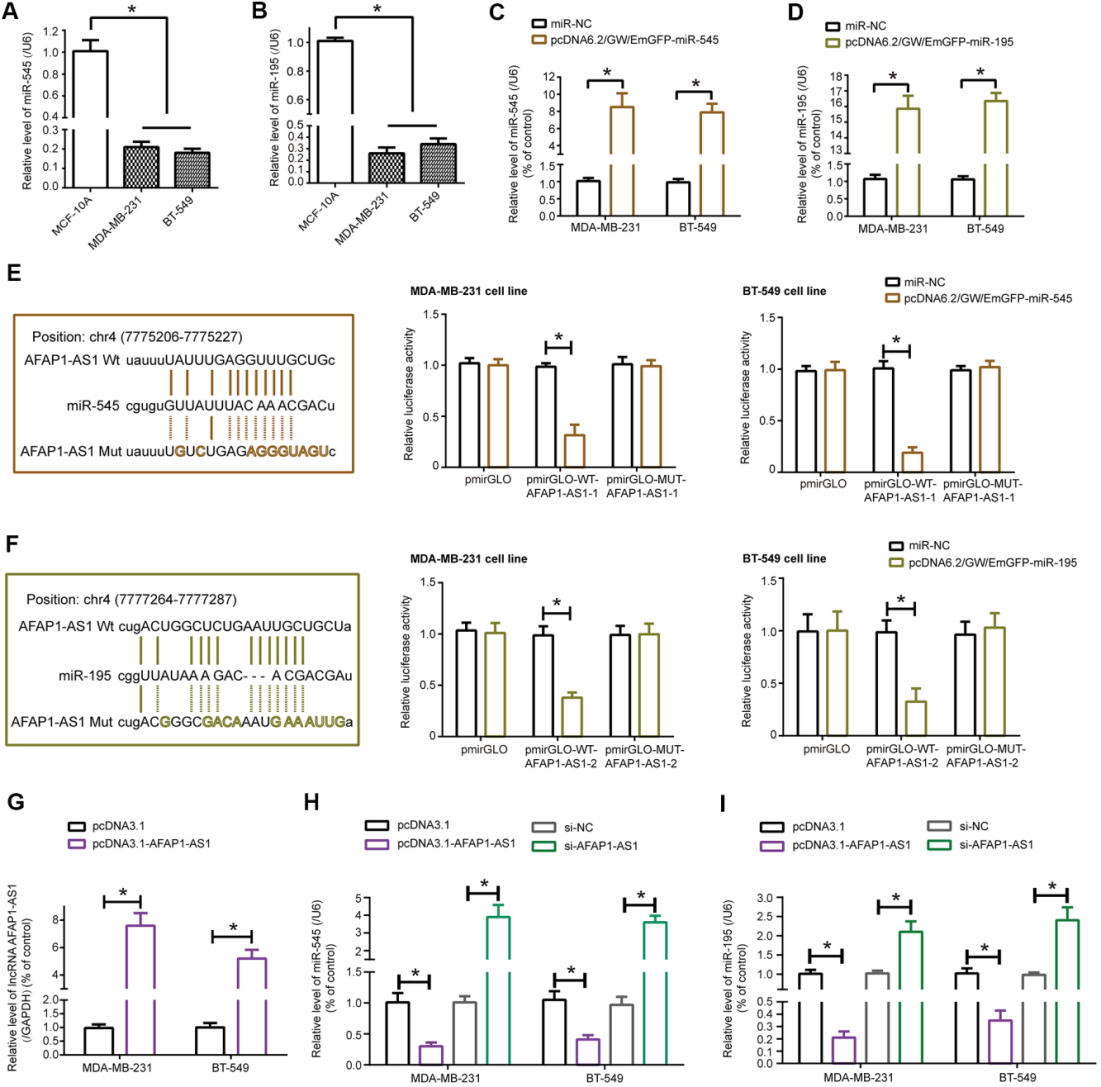
**MiR-545-3p and miR-195 were sponged and modified by lncRNA AFAP1-AS1 in triple-negative breast cancer (TNBC) cells.** (**A**, **B**) Expressions of miR-545-3p (**A**) and miR-195 (**B**) were lower in MDA-MB-231 and BT-549 cell lines than in MCF-10A cell line. *: *P*<0.05. (**C**, **D**) Expressions of miR-545-3p (**C**) and miR-195 (**D**) were boosted in MDA-MB-231 and BT-549 cell lines after respective transfections of pcDNA6.2/GW/EmGFP-miR-545 and pcDNA6.2/GW/EmGFP-miR-195. *: *P*<0.05. (**E**, **F**) MiR-545-3p (**E**) and miR-195 (**F**) were sponged by lncRNA AFAP1-AS1 in certain targets, and MDA-MB-231 and BT-549 cell lines of pmirGLO-WT-lncRNA AFAP1-AS1+pcDNA6.2/GW/EmGFP-miR-545/miR-195 group were associated with weaker luciferase activity than TNBC cell lines of pmirGLO-MUT-lncRNA AFAP1-AS1+pcDNA6.2/GW/EmGFP-miR-545/miR-195 group. *: *P*<0.05. (**G**) LncRNA AFAP1-AS1 expression in MDA-MB-231 and BT-549 cell lines was determined when pcDNA3.1-lncRNA AFAP1-AS1 was transfected. *: *P*<0.05. (**H**, **I**) Expressions of miR-545 (**H**) and miR-195 (**I**) were detected among MDA-MB-231 and BT-549 cell lines transfected by pcDNA3.1, pcDNA3.1-lncRNA AFAP1-AS1, si-NC and si-lncRNA AFAP1-AS1. *: *P*<0.05.

MiRNAs expected to be sponged by lncRNA AFAP1-AS1, drawn from ENCORI online database (http://starbase.sysu.edu.cn/agoClipRNA.php?source=lncRNA&flag=target&clade=mammal&genome=human&assembly=hg19&miRNA=all&clipNum=1&deNum=0&panNum=0&target=AFAP1-AS1) (Supplementary Figure 2) [26], were determined in MCF-10A, MDA-MB-231 and BT-549 cell lines (Supplementary Figure 3A and Figure 2A, 2B), which revealed that miR-545-3p, miR-195, miR-424-5p, miR-497-5p, miR-216a-5p, miR-190a-5p and miR-655-3p were dramatically under-expressed in MDA-MB-231 and BT-549 cell lines as relative to MCF-10A cell line (all *P*<0.05). Furthermore, expressions of miRNAs, including miR-545-3p (Figure 2C) and miR-195 (Figure 2D), were remarkably elevated in MDA-MB-231 and BT-549 cell lines after transfection of their respective pcDNA6.2/GW/EmGFP forms (all *P*<0.05) (data not shown for other miRNAs). Relationships between lncRNA AFAP1-AS1 and miRNAs were evaluated based on luciferase reporter gene assay (Supplementary Figure 3B), which demonstrated that miR-545-3p and miR-195 were probably sponged by lncRNA AFAP1-AS1 in both MDA-MB-231 and BT-549 cell lines, since that the luciferase activity of MDA-MB-231 and BT-549 cell lines became weak in the pmirGLO-WT-lncRNA AFAP1-AS1+pcDNA6.2/GW/EmGFP-miR-545/miR-195 group as compared with pmirGLO-MUT-lncRNA AFAP1-AS1+pcDNA6.2/GW/EmGFP-miR-545/miR-195 group and pmirGLO-WT-lncRNA AFAP1-AS1+miR-NC group (*P*<0.05) (Figure 2E, 2F).

Furthermore, miRNAs were monitored in MDA-MB-231 and BT-549 cell lines transfected by si-lncRNA AFAP1-AS1, and the results insinuated that miR-545-3p and miR-195 were both markedly up-regulated in MDA-MB-231 and BT-549 cell lines of si-lncRNA AFAP1-AS1 group as relative to si-NC group (*P*<0.05) (Supplementary Figure 4). To emphasize the influence of lncRNA AFAP1-AS1 on miR-545-3p and miR-195, pcDNA3.1-lncRNA AFAP1-AS1 was transfected so as to raise lncRNA AFAP1-AS1 expression in TNBC cell lines (*P*<0.05) (Figure 2G), through which we discovered that expressions of miR-545 and miR-195 were significantly inhibited in case lncRNA AFAP1-AS1 was over-expressed (*P*<0.05) (Figure 2H, 2I). Not only that, it was speculated by miRPathDB database (https://mpd.bioinf.uni-sb.de/overview.html) that genes subjected to target of miR-195 and miR-545 were enriched in tumorigenesis-related KEGG pathways (Supplementary Figure 5), further stressing that miR-195 and miR-545 were vital targets of lncRNA AFAP1-AS1 in TNBC.

### MiR-545-3p hindered lncRNA AFAP1-AS1-reinforced chemo-resistance, proliferation, migration and invasion of TNBC cells

**Figure 3:**
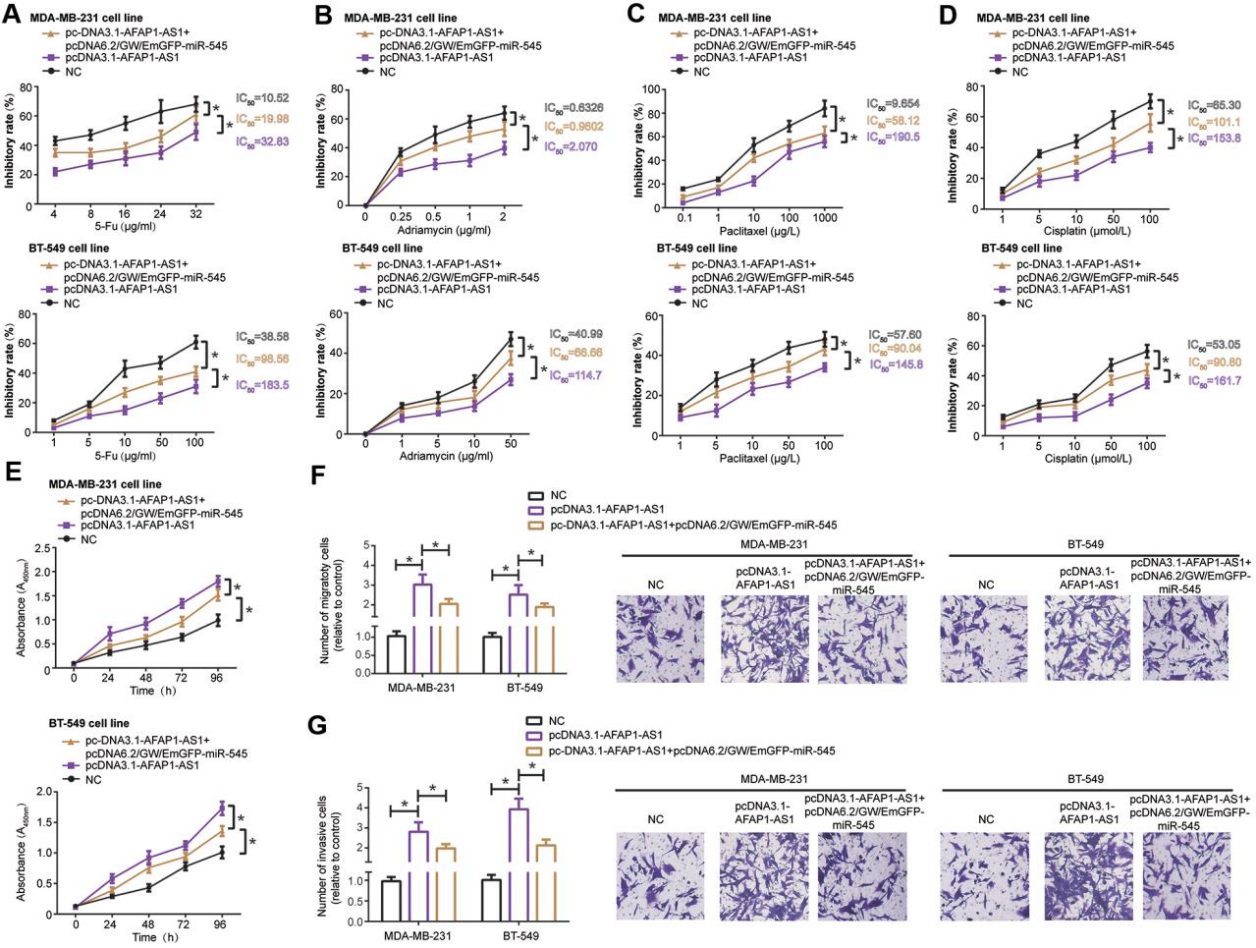
MiR-545-3p disturbed the influence of lncRNA AFAP1-AS1 on chemo-resistance (**A**–**D**), proliferation (**E**), migration (**F**) and invasion (**G**) of triple-negative breast cancer (TNBC) cells. *: *P*<0.05.

MDA-MB-231 and BT-549 cell lines transfected by pcDNA3.1-lncRNA AFAP1-AS1 demonstrated stronger resistance against 5-Fu (Figure 3A), adriamycin (Figure 3B), paclitaxel (Figure 3C) and cisplatin (Figure 3D) than TNBC cell lines transfected by none (all *P*<0.05), and pcDNA3.1-lncRNA AFAP1-AS1 combined with pcDNA6.2/GW/EmGFP-miR-545-3p markedly enhanced chemo-resistance of MDA-MB-231 and BT-549 cells in comparison to pcDNA3.1-lncRNA AFAP1-AS1 transfection alone (all *P*<0.05) (Figures 3A–3D). Moreover, proliferation (Figure 3E), migration (Figure 3F) and invasion (Figure 3G) of MDA-MB-231 and BT-549 cells were reinforced in the pcDNA3.1-lncRNA AFAP1-AS1 group as compared with NC group (all *P*<0.05), however, these malignant behaviors were undermined in the pcDNA3.1-lncRNA AFAP1-AS1+pcDNA6.2/GW/EmGFP-miR-545 group as opposed to pcDNA3.1-lncRNA AFAP1-AS1 group (all *P*<0.05) (Figure 3E–3G).

**Figure 4:**
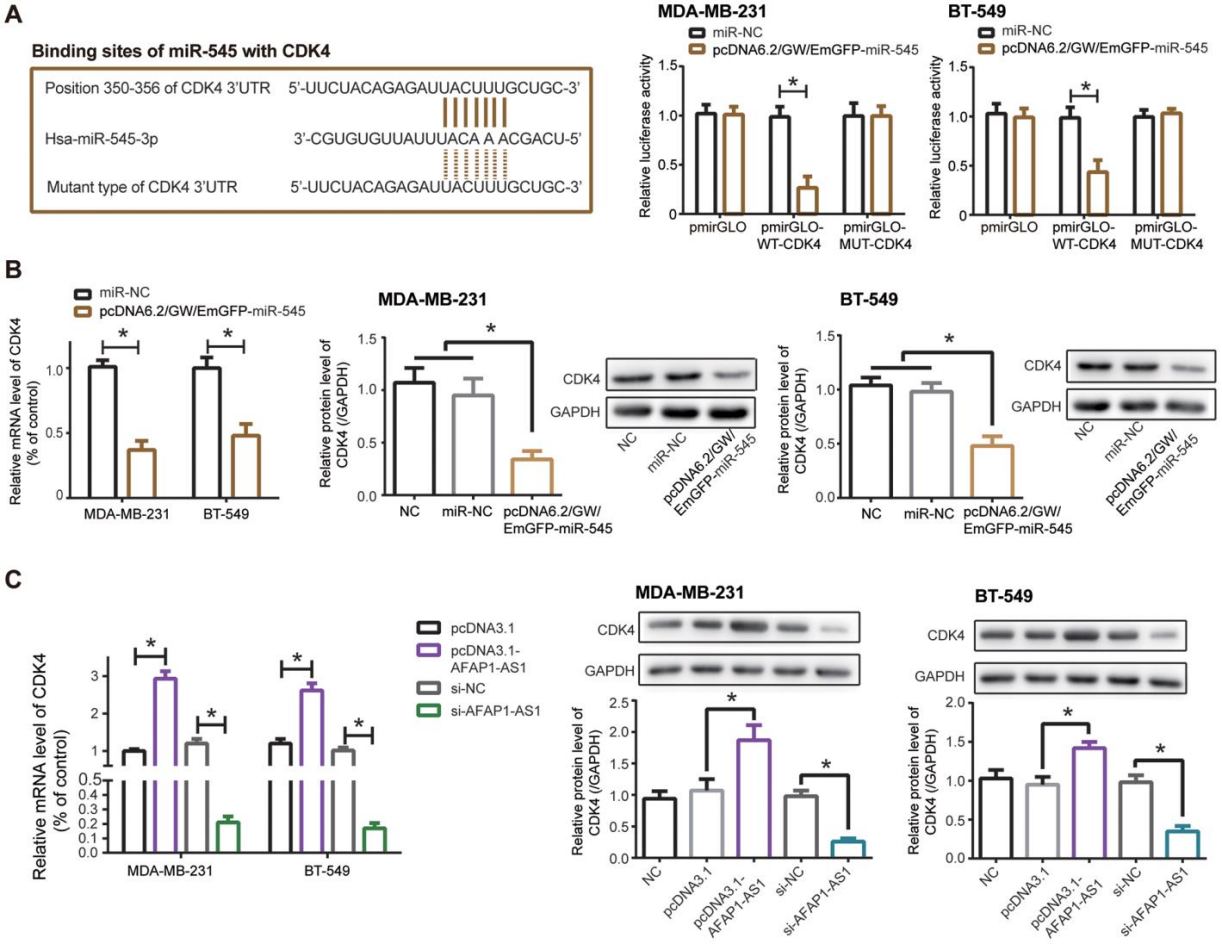
**CDK4 was regulated by lncRNA AFAP1-AS1 and miR-545 in triple-negative breast cancer (TNBC) cells.** (**A**) CDK4 was targeted by miR-545 in certain sites, and luciferase activity of MDA-MB-231 and BT-549 cell lines in the pmirGLO-WT-CDK4+pcDNA6.2/GW/EmGFP-miR-545 group was decreased as relative to pmirGLO-MUT-CDK4+pcDNA6.2/GW/EmGFP-miR-545 group. *: *P*<0.05. (**B**, **C**) Both mRNA and protein levels of CDK4 in MDA-MB-231 and BT-549 cell lines were modulated by pcDNA6.2/GW/EmGFP-miR-545 (B) and pcDNA3.1-lncRNA AFAP1-AS1/si-lncRNA AFAP1-AS1 (**C**). *: *P*<0.05.

CDK4, an indicator of TNBC progression [27], was targeted by miR-545 in MDA-MB-231 and BT-549 cell lines (Figure 4A), and luciferase activity of MDA-MB-231 and BT-549 cells was decreased in the pmirGLO-WT-CDK4+pcDNA6.2/GW/EmGFP-miR-545 group as relative to pmirGLO-MUT-CDK4+pcDNA6.2/GW/EmGFP-miR-545 group and pmirGLO-WT-CDK4+miR-NC group (*P*<0.05). Furthermore, mRNA and protein levels of CDK4 was down-regulated in TNBC cell lines after transfection of pcDNA6.2/GW/EmGFP-miR-545, when compared with NC group and miR-NC group (*P*<0.05) (Figure 4B).

Silencing of lncRNA AFAP1-AS1 also observably reduced mRNA and protein levels of CDK4 in comparison to si-NC group (*P*<0.05), while mRNA and protein levels of CDK4 were boosted in pcDNA3.1-lncRNA AFAP1-AS1 group as relative to pcDNA3.1 group (*P*<0.05) (Figure 4C). Together, miR-545/CDK4 axis was critical for lncRNA AFAP1-AS1-involved TNBC pathogenesis.

### MiR-195 reversed contribution of lncRNA AFAP1-AS1 to chemo-resistance, proliferation, migration and invasion of TNBC cells

**Figure 5:**
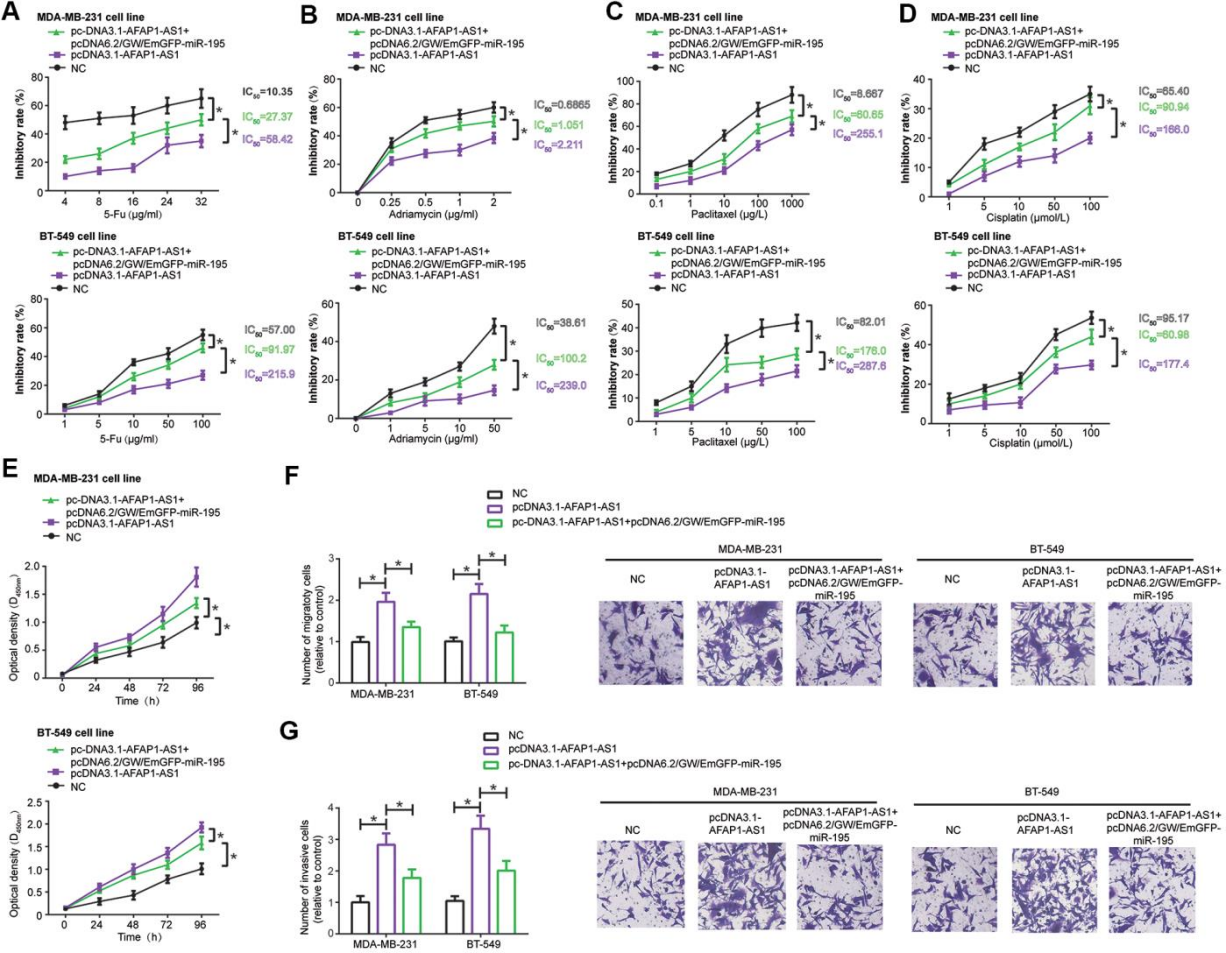
MiR-195 interfered with the influence of lncRNA AFAP1-AS1 on drug-resistance (**A**–**D**), proliferation (**E**), migration (**F**) and invasion (**G**) of triple-negative breast cancer (TNBC) cells. *: *P*<0.05.

MDA-MB-231 and BT-549 cells in the pcDNA3.1-lncRNA AFAP1-AS1+pcDNA6.2/GW/EmGFP-miR-195 group became less resistant to docetaxel (Figure 5A), adriamycin (Figure 5B), paclitaxel (Figure 5C) and cisplatin (Figure 5D) than TNBC cells in the pcDNA3.1-lncRNA AFAP1-AS1 group (all *P*<0.05). Likewise, TNBC cells in the pcDNA3.1-lncRNA AFAP1-AS1+pcDNA6.2/GW/EmGFP-miR-195 group were restrained from proliferating (Figure 5E), migrating (Figure 5F) and invading (Figure 5G), as opposed to cells in the pcDNA3.1-lncRNA AFAP1-AS1 group (all *P*<0.05).

**Figure 6:**
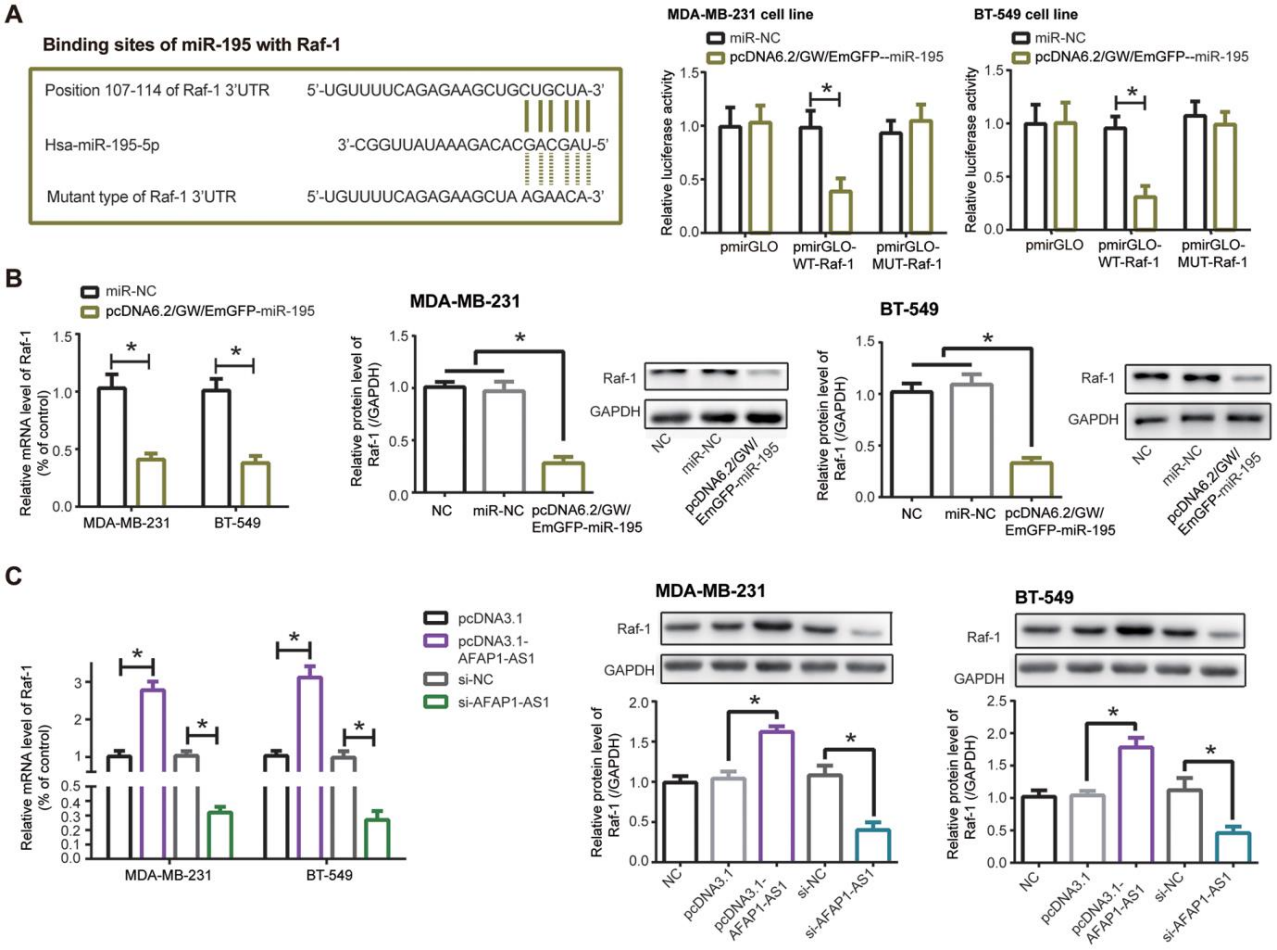
**Raf-1 was implicated in the impact of lncRNA AFAP1-AS1/miR-195 axis on triple-negative breast cancer (TNBC) cells.** (**A**) miR-195 targeted Raf-1 in certain sites, and MDA-MB-231/BT-549 cell lines in the pmirGLO-WT-Raf-1+pcDNA6.2/GW/EmGFP-miR-195 group showed decreased luciferase activity in comparison to pmirGLO-MUT-Raf-1+pcDNA6.2/GW/EmGFP-miR-195 group. *: *P*<0.05. (**B**, **C**) Raf-1 expression in MDA-MB-231 and BT-549 cell lines was affected by pcDNA6.2/GW/EmGFP-miR-195 (**B**) and pcDNA3.1-lncRNA AFAP1-AS1/si-lncRNA AFAP1-AS1 (**C**) at mRNA and protein levels. *: *P*<0.05.

When compared with pmirGLO-MUT-Raf-1+pcDNA6.2/GW/EmGFP-miR-195 group and pmirGLO-MUT-Raf-1+miR-NC group, co-transfection of pmirGLO-WT-Raf-1 and pcDNA6.2/GW/EmGFP-miR-195 engendered a dramatic reduction of luciferase activity in MDA-MB-231 and BT-549 cells (*P*<0.05), implying that miR-195 targeted Raf-1, a TNBC-specific biomarker [28], in specific sites (Figure 6A). What’s more, mRNA and protein levels of Raf-1 were lowered in MDA-MB-231 and BT-549 cells transfected by pcDNA6.2/GW/EmGFP-miR-195, as compared with NC group and miR-NC group (*P*<0.05) (Figure 6B). And Raf-1 expression ascended markedly in the pcDNA3.1-lncRNA AFAP1-AS1 group as relative to pcDNA3.1 group (*P*<0.05), yet declined notably in the si-lncRNA AFAP1-AS1 group in comparison to si-NC group (*P*<0.05) (Figure 6C). Taken together, miR-195/Raf-1 axis participated in lncRNA AFAP1-AS1-mediated TNBC etiology.

### Formononetin held back proliferation, migration and invasion of TNBC cells by disturbing lncRNA AFAP1-AS1-miR-545/miR-195 axis

**Figure 7:**
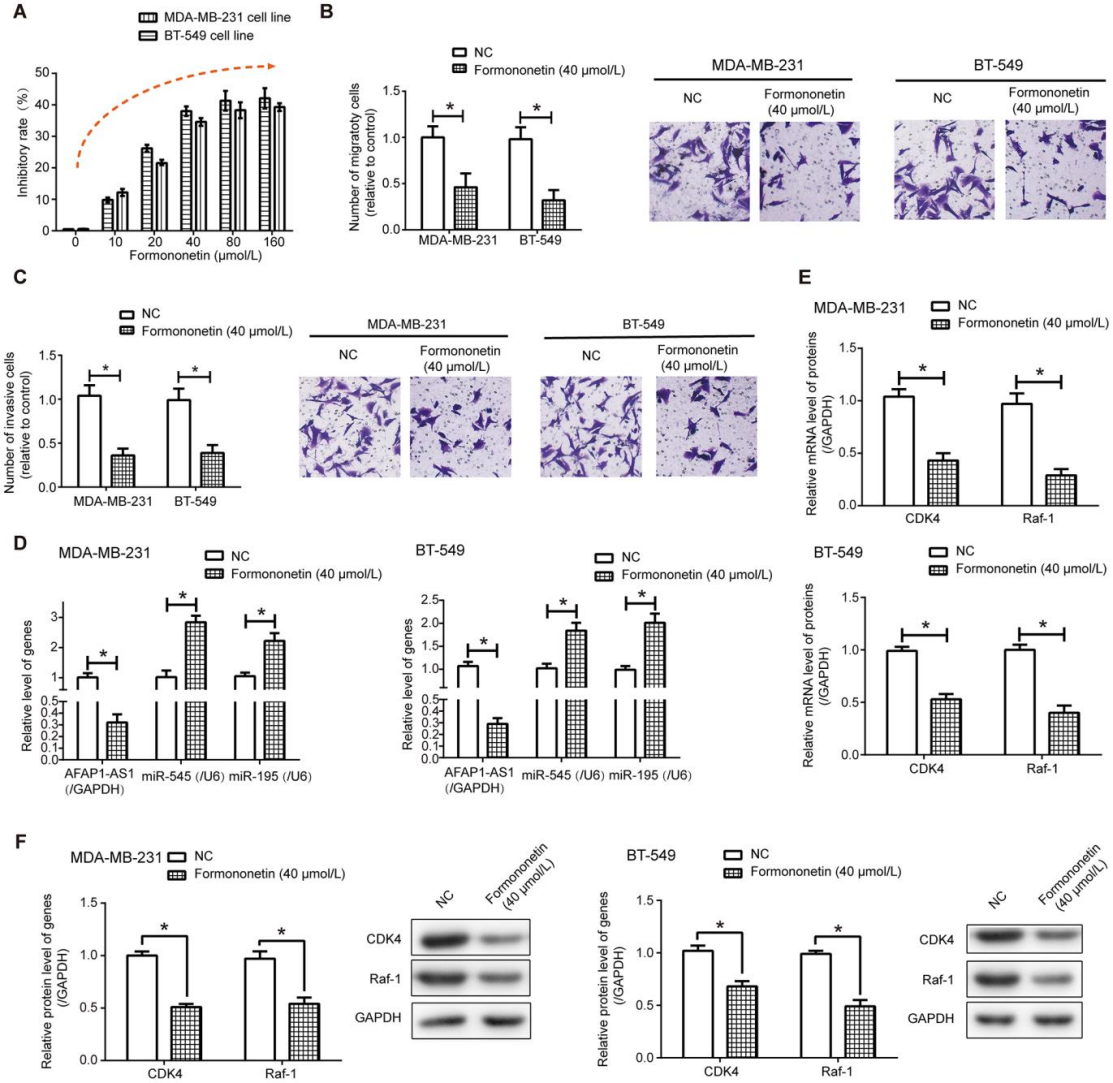
**Formononetin undermined activities of triple-negative breast cancer (TNBC) cells via depression of lncRNA AFAP1-AS1-miR-545/miR-195 axis.** (**A**–**C**) Formononetin postponed proliferation (**A**), migration (**B**) and invasion (**C**) of MDA-MB-231 and BT-549 cell lines. *: *P*<0.05. (**D**) Expressions of lncRNA AFAP1-AS1, miR-545 and miR-195 were detected in MDA-MB-231 and BT-549 cell line after formononetin exposure. *: *P*<0.05. (**E**, **F**) Both mRNA (**E**) and protein (**F**) levels of Raf-1 and CDK4 were measured in MDA-MB-231 and BT-549 cell lines treated by formononetin. *: *P*<0.05.

After exposure to formononetin, proliferation of MDA-MB-231 and BT-549 cell lines was undermined dose- dependently (*P*<0.05), and this inhibition reached a maximum when formononetin concentration was designated as 40 μmol/L and 80 μmol/L (Figure 7A). Formononetin treatment at the concentration of 40 μmol/L also potently retarded migration (Figure 7B) and invasion (Figure 7C) of MDA-MB-231 and BT-549 cell lines (*P*<0.05).

Additionally, lncRNAs that were differentially expressed between MDA-MB-231 cell line and MDA-MB-231/DDP cell line (Supplementary Table 1), as well as lncRNAs documented to involve in TNBC chemo-resistance, including lncRNA H19 [29], Linc00152 [30], lncRNA SPRY4-IT1 [31], lncRNA FTH1P3 [32], linc ROR [33], lncRNA XIST [34], lncRNA CASC2 [35], lncRNA DLX6-AS1 [36] and lncRNA SNHG15 [37], were measured in formononetin-treated MDA-MB-231 and BT-549 cells (Supplementary Figure 6). We noticed that lncRNA AFAP1-AS1 expression in MDA-MB-231 and BT-549 cell lines was prominently decreased under the influence of 40 μmol/L formononetin (*P*<0.05) (Figure 7D and Supplementary Figure 6). LncRNA AFAP1-AS1-sponged miRNAs, conjectured from ENCORI online database [26], were also detected (Supplementary Figure 7), which revealed that expressions of miR-545-3p and miR-195 were significantly enhanced in formononetin-treated MDA-MB-231 and BT-549 cells as relative to NC group (*P*<0.05) (Figure 7D). More than that, exposure to 40 μmol/L formononetin gave rise to prominent decreases of CDK4 and Raf-1 at both mRNA (Figure 7E) and protein (Figure 7F) levels, whether in MDA-MB-231 cell line or in BT-549 cell line (*P*<0.05).

## DISCUSSION

Early recurrence, swift progression and poor prognosis constitute major obstacles to successful treatment of TNBC [38, 39], so in-depth understanding of TNBC etiology is required, and formulating treatment strategies that work for TNBC has become a necessity.

Multitudes of researchers have gradually realized how closely lncRNAs, including oncogenic lncRNA HOTAIR [40], lncRNA MALAT1 [41], lncRNA LSINCT5 [42], lncRNA H19 [43] and lncRNA BC200 [44], as well as protective lncRNA XIST [45] and lncRNA GAS5 [46], were intertwined with BC onset and deterioration. It was also corroborated that doxorubicin-sensitivity of MCF-7 cell line was rescued in the presence of high-level lncRNA Adriamycin Resistance Associated (ARA) [47], while lncRNA Breast Cancer Anti-Estrogen Resistance 4 (BCAR4) functioned to strengthen tamoxifen-resistance of MCF-7 cell line and ZR-75-1 cell line [48]. Partly aligning with the speculation of Zhang et al. [15], we concluded that tracking expressional trend of lncRNA AFAP1-AS1 might help to determine TNBC onset and to predict TNBC prognosis of a Chinese population (Supplementary Figure 1), which, however, failed to go for patients of other BC subtypes (Supplementary Tables 2, 3). In spite of this, whether lncRNA AFAP1-AS1 maintained this specificity in populations of other ethnicities and scales awaited validations. Of note, silencing of lncRNA AFAP1-AS1 tended to dampen malignant behaviors of TNBC cells (Figure 1B, 1G–1I), which, from the molecular standpoint, accounted for why lncRNA AFAP1-AS1 facilitated negative clinical outcomes in TNBC patients (Supplementary Figure 1). Virtually, besides TNBC, oncogenesis of lncRNA AFAP1-AS1 was also identifiable in neoplasms including esophageal adenocarcinoma, gallbladder cancer, gastric cancer, cholangiocarcinoma, colorectal cancer and pancreatic ductal adenocarcinoma [49–51]. It might be due to these tumor-promoting actions that lncRNA AFAP1-AS1 powerfully heightened cisplatin-resistance of esophageal squamous cell carcinoma [52]/laryngeal carcinoma cells [53], paclitaxel-resistance of prostate cancer cells [54], 5-Fu-/cisplatin-resistance of non-small cell lung cancer cells [55], as well as 5-Fu/adriamycin/paclitaxel/cisplatin-resistance of TNBC cells manifested in this study (Figure 1C–1F). Nonetheless, this investigation hardly compared lncRNA AFAP1-AS1 expression between TNBC patients who accepted chemotherapy and people who refused drug treatments, so that the clinical linkage of lncRNA AFAP1-AS1 with TNBC chemo-resistance was unavailable.

Inspired by the classical ceRNA hypothesis [56], scholars became increasingly aware of the strong connection of lncRNA AFAP1-AS1 with carcinogenesis-deactivating miRNAs. For example, lncRNA AFAP1-AS1 urged metastasis of esophageal cancer cells by binding to miR-26a and then augmenting ATF2 expression [57]. As far as esophageal squamous carcinoma was concerned, lncRNA AFAP1-AS1 decreased suppressive influence of miR-498 on protein levels of VEGFA, thereby delaying apoptosis of the tumor cells [58]. Beyond these miRNAs, we discovered that miR-545 and miR-195 were crucial targets of lncRNA AFAP1-AS1 in TNBC (Supplementary Figures 2, 3B, 4 and Figure 2E–2I), and they attenuated lncRNA AFAP1-AS1-fortified proliferation, metastasis and drug resistance of TNBC cells (Figures 3, 5). Regarding miR-545-3p, apart from under-expression in TNBC cells (Figure 2A), it debilitated growth of lung cancer cells [59], pancreatic cancer cells [60], cervical cancer cells [61] and colon adenocarcinoma cells [62], nevertheless, Liu et al. found it paradoxical that proliferation of hepatocellular carcinoma cells was drastically motivated when *in-vitro* miR-545 level was intentionally heightened [63]. This contradiction might result from discrepant pathological attributes that miR-545 exhibited in entirely different neoplasms. More than that, we suspected that CDK4, a component necessitated for cell-cycle progression by activating E2F and CyclinE [64–66], was of significance to elaborate lncRNA AFAP1-AS1/miR-545-3p-involved TNBC development and chemo-resistance (Figures 4, 5), and the miR-545/CDK4 axis has been underlined in explaining etiologies of colorectal cancer [67] and lung cancer [59]. For another, miR-195, whose expression was markedly down-regulated in colon cancer [68], gastric cancer [69], bladder cancer [70], cervical cancer [71] and TNBC (Figure 2B), also conferred incremental chemo-sensitivity in tumors, including glioblastoma [72], colorectal cancer [73] and TNBC herein (Figure 5). We further argued that Raf-1, whose phosphorylation of ERK remarkably stimulated growth and metastasis of TNBC cells [74], was core to lncRNA AFAP1-AS1/miR-195-mediated TNBC progression, allowing for its level change in TNBC cell lines after stimulation by lncRNA AFAP1-AS1 and miR-195 (Figure 6). Collectively, this investigation newly uncovered that miR-545-3p/CDK4 axis and miR-195/Raf-1 axis participated in restoring contribution of lncRNA AFAP1-AS1 to TNBC development.

Additionally, formononetin, a Chinese herb, was expected to diminish malignant activities of TNBC cells (Figure 7A–7C) [19] by repressing lncRNA AFAP1-AS1-led miRNA axes (Supplementary Figures 6, 7 and Figure 7D, 7E), which widened current cognitions about how formononetin leveraged molecular networks, in addition to MAPK pathway [75] and JAK/STAT pathway [76], to mitigate TNBC exacerbation. Moreover, researches so far mostly highlighted that formononetin halted carcinogenesis, including laryngeal cancer [77], nasopharyngeal cancer [78], glioma [79] and multiple myeloma [80], by squinting tumor cells to apoptosis and by forbidding them from metastasizing [81]. However, formononetin also held potential in overcoming hyper-inflammation [82], which was relevant to unfavorable TNBC prognosis [83], but whether formononetin combatted TNBC development in an inflammation-dependent mode was unvalidated here.

## CONCLUSIONS

**Figure 8:**
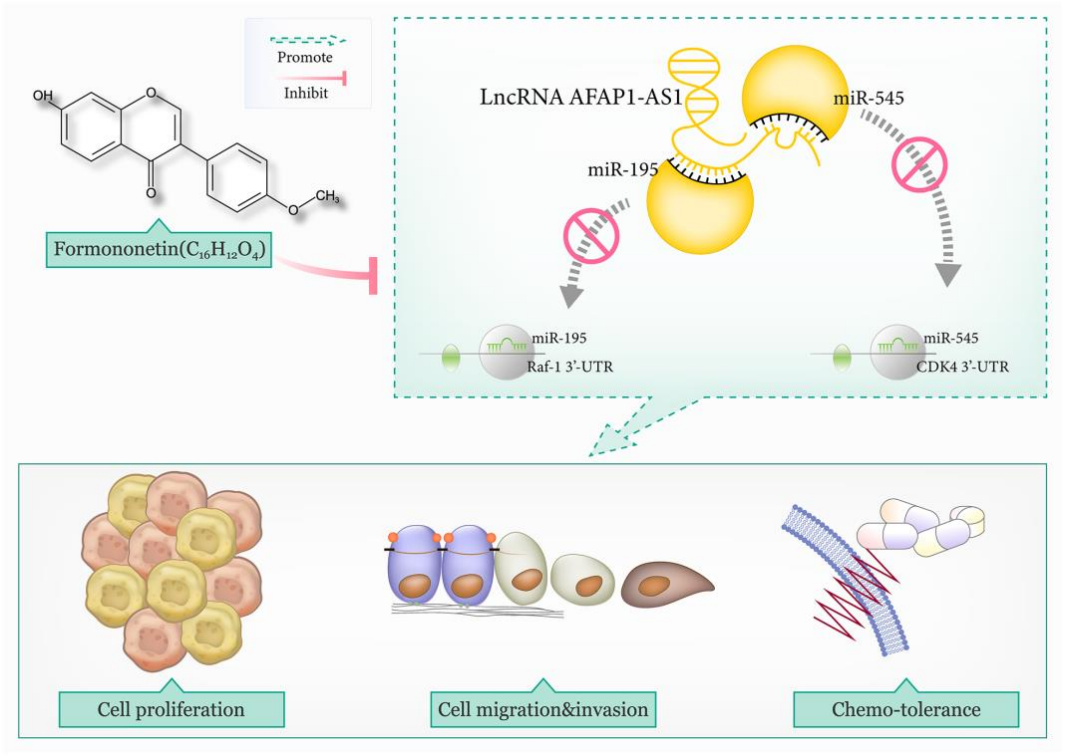
**The mechanism map illustrated that lncRNA AFAP1-AS1 promoted triple-negative breast cancer (TNBC) progression and chemo-resistance by disturbing the interaction of miR-195 with Raf-1 and that of miR-545 with CDK4.** However, formononetin antagonized TNBC malignancy by lessening the effect of lncRNA AFAP1-AS1-guided miR-545/CDK4 axis and miR-195/Raf-1 axis on TNBC cells.

Collectively, formononetin exerted anti-TNBC function by reducing the influence of lncRNA AFAP1-AS1 on miR-545-3p/CDK4 axis and miR-195/Raf-1 axis, which were associated with TNBC exacerbation and chemoresistance (Figure 8). There were, however, a couple of deficiencies in the experimental design. For one thing, although tumor growth in TNBC-bearing mice models, was inhibited by formononetin at the concentration of 80 mg/kg (Supplementary Table 4), along with decreased lncRNA AFAP1-AS1 level and increased miR-545-3p/miR-195 level in the tumor tissues (Supplementary Figure 8), joint effects of formononetin, si-lncRNA AFAP1-AS1 and miR-545-3p/miR-195 mimic on tumor growth in the mice models were not studied. For another, considering that single-target therapy led to smaller objective response rates than multiple-target therapy in terms of treating solid tumors [84, 85], combined application of molecular targets and formononetin might be viable for TNBC treatment, but this point was not clinically supported.

## MATERIALS AND METHODS

### Cell culture

TNBC cell lines (i.e. MDA-MB-231 and BT-549) and normal human mammary epithelial cell line (i.e. MCF-10A), purchased from American Type Culture Collection (ATCC, USA), were cultured in RPMI-1640 medium (Gibco, USA) which incorporated 10% (v/v) fetal bovine serum (FBS), 100 U/mL streptomycin and 100 U/mL penicillin. After overnight cultivation in 5% CO_2_ at 37° C, MDA-MB-231 and BT-549 cell lines at the logarithmic growth phase were reserved.

### Cell transfection

When confluency of MDA-MB-231 and BT-549 cells reached nearly 80%, pcDNA3.1-lncRNA AFAP1-AS1 (Invitrogen, USA), lncRNA AFAP1-AS1-siRNA (5’-CCTATCTGGTCAACACGTA-3’, Genepharma, China), si-negative control (NC) (sense: 5’-GCGACGAUCUGCCUAAGA-3’, anti-sense: 5’-AUCUUAGGCAGAUCGUCG-3’, Invitrogen, USA), pcDNA6.2/GW/EmGFP-miRNAs (Sangon, China) and pcDNA6.2/GW/EmGFP-miR-NC (named as miR-NC, Sangon, China) were, respectively, transfected into the TNBC cell lines for 48 h, aided by Lipofectamine 2000^TM^ reagent (Invitrogen, USA). The experiments were implemented with more than 3 replicates.

### MTT assay to evaluate chemo-resistance of TNBC cells

MDA-MB-231 and BT-549 cell lines, inoculated into 96-well plates at the density of 2500/well, were disposed by gradient concentrations of 5-Fu ((Beijing Zhongshan Jinqiao Biotechnology, China), adriamycin (Zhejiang HISUN Pharmaceuticals, China), paclitaxel (Sino-American Shanghai Squibb Pharmaceuticals, China) and cisplatin (Beijing Zhongshan Jinqiao Biotechnology, China) separately for 48 h. Subsequently, TNBC cells in each well were managed by 15 μl MTT at the concentration of 5 mg/ml (Sinopharm Chemical Reagent Corporation, China) for 4 hours, and then 150 μl DMSO (BD, USA) was dropped into each well to mix with the TNBC cells for around 10 min. Absorbance at 490 nm (A490) of TNBC cells under each treatment was measured by virtue of full-wavelength microplate reader (model: 550, Forma Scientific, USA). Inhibitory rate (%) of chemo-drugs on growth of TNBC cells was assessed based on the formula of (1-A490_drug group_/A490_control group_) × 100%, and half maximal inhibitory concentration (IC50) values were calculated. The experiments were conducted with ≥ 3 replicates.

### Cell treatment by formononetin

MDA-MB-231 and BT-549 cells adjusted to the density of 5×10^4^/ml were seeded into 96-well culture plates, and they were starved in serum-free medium for 24 h. Afterwards, the TNBC cells were exposed to 10 μmol/L, 20 μmol/L, 40 μmol/L, 80 μmol/L and 160 μmol/L formononetin (batch number: 111703-200603, China National Institute for Food and Drug Control), respectively, for 24 h. The experiments were repeated for ≥ 3 times.

### Real-time quantitative PCR (RT-PCR)

BC tissues frozen within liquid nitrogen, as well as BC cell lines, were lysed after addition of 1ml TRIzol reagent (Invitrogen, USA), through which total RNAs were isolated. Concentration and purity of the RNAs were assessed using an ultraviolet (UV) spectrophotometer (model: NanoDropND-1000, NanoDrop Technologies, USA), and RNA samples whose A260/A280 ratio lied between 1.8 and 2.1 were reserved. Reverse transcription of the RNAs was implemented following procedures described in PrimeScript^TM^ RT Master Mix kit (Takara, Japan) or miScript II RT kit (Qiagen, Germany), and the obtained cDNAs were amplified by employing real-time PCR kit (Takara, Japan) or miScript SYBR® Green PCR kit (Qiagen, Germany). Primers for genes were ordered in Supplementary Table 5, and their relative expression was normalized by means of 2^-ΔΔCt^ method [86]. These experiments were repeated for at least 3 times.

### Western blotting

After denaturation at 105° C for 5 min, total protein extracted from BC tissues and cell lines was separated by electrophoresis, successively experiencing 1) 80 V for 2~3 h and 2) 100 V for 90 min. With usage of electrophoretic transfer apparatus (model: Mini Trans-Blot, Bio-Rad, USA), proteins on the gel were transferred onto polyvinylidene fluoride (PVDF) membrane through wet method. Afterwards, the membrane was placed within 10 ml blocking buffer (i.e. 2% skim milk) for 1 h, and protein samples were incubated by primary antibodies (rabbit-anti-human, Abcam, USA) against CDK4 (1: 2000, Catalog No: ab108357), Raf-1 (1: 2000, Catalog No: ab137435) and GAPDH (1: 10000, Catalog No: ab181602) at 4° C for overnight. Then the products were incubated by goat anti-rabbit IgG H&L labelled by horseradish peroxidase (HRP) (1:5000, Catalog No: ab205718, Abcam, USA) at room temperature for 2 h. Development of protein samples was carried out by adopting chemiluminescence (ECL) (Pierce, USA), and gray values of protein bands were determined through utilization of Image-Pro Plus software (Media Cybernetics, USA). The experiments were carried out for at least 3 times.

### CCK-8 assay

MDA-MB-231 and BT-549 cells were seeded into 96-well plates at the density of 3000 cells per well. After overnight culture, 10 μl CCK-8 reagent (Dojindo, Japan) was supplemented gently into each well at the time point of 0 h. After cultivation at 37° C for 24 h, 48 h, 72 h and 96 h, absorbance (A) of TNBC cells in each well was monitored at 450 nm on the microplate reader (Bio-Rad, USA). These experiments were performed for at least 3 times.

### Transwell assay

### Cell migration

MDA-MB-231 and BT-549 cells at the concentration of 1×10^5^/ml were paved onto the upper Transwell chamber (Corning Costar, USA), and 600 μl DMEM medium that contained 10% FBS was poured into the lower transwell chamber (Corning Costar, USA). After routine culture for 24 h, the TNBC cells were stained by 0.1% crystal violet (Solarbio Life Sciences, China), thereafter photographs were taken under optical microscope (Olympus, USA). The experiments were undertaken with ≥ 3 replicates.

### Cell invasion

Procedures of cell invasion assay were mostly consistent with those of cell migration assay, except that Matrigel diluted by DMEM (ratio: 1/6) was added into the upper Transwell chamber (Corning Costar, USA), after which suspension of MDA-MB-231/BT-549 cells and DMEM medium were supplemented.

### Dual luciferase reporter gene assay

LncRNA AFAPA-AS1 and RAF1 fragments that contained miR-195-binding sites, drawn from Encyclopedia of RNA Interactomes (ENCORI) online database (http://starbase.sysu.edu.cn/) [26], were amplified through conduction of PCR, in a bid to construct wide types of lncRNA AFAPA-AS1 (i.e. WT-lncRNA AFAP1-AS1-1) and Raf-1 (i.e. WT-Raf-1). Simultaneously, mutant types of lncRNA AFAPA-AS1 (i.e. MUT-lncRNA AFAP1-AS1-1) and Raf-1 (i.e. MUT-Raf-1) were produced by mutating miR-545-binding sites in lncRNA AFAPA-AS1 and RAF1. After that, WT-lncRNA AFAP1-AS1-1, WT-Raf-1, MUT-lncRNA AFAP1-AS1-1 and MUT-Raf-1 were, respectively, connected to pmirGLO vector (Promega, USA), in order to establish pmirGLO-WT-lncRNA AFAP1-AS1-1, pmirGLO-WT-Raf-1, pmirGLO-MUT-lncRNA AFAP1-AS1-1 and pmirGLO-MUT-Raf-1. With respect to miR-195, lncRNA AFAPA-AS1 and CDK4 fragments that possessed miR-195-targeting sites were reserved to construct pmirGLO-WT-lncRNA AFAP1-AS1-2 and pmirGLO-WT-CDK4, while pmirGLO-MUT-lncRNA AFAP1-AS1-2 and pmirGLO-MUT-CDK4 were established via mutation of their respective miR-195-targeting sites. Subsequently, MDA-MB-231 and BT-549 cells of logarithmic growing phase were inoculated into 96-well plates at the density of 4×10^3^/well, and they were transfected by 1) pcDNA6.2/GW/EmGFP-miR-545+pmirGLO-WT-lncRNA AFAP1-AS1-1/pmirGLO-WT-Raf-1, 2) pcDNA6.2/GW/EmGFP-miR-545+pmirGLO-MUT-lncRNA AFAP1-AS1-1/pmirGLO-MUT-Raf-1, 3) miR-NC+pmirGLO-WT-lncRNA AFAP1-AS1-1/pmirGLO-WT-Raf-1, 4) miR-NC+pmirGLO-MUT-lncRNA AFAP1-AS1-1/pmirGLO-MUT-Raf-1, 5) pcDNA6.2/GW/EmGFP-miR-195+pmirGLO-WT-lncRNA AFAP1-AS1-2/pmirGLO-WT-CDK4, 6) pcDNA6.2/GW/EmGFP-miR-195+pmirGLO-MUT-lncRNA AFAP1-AS1-2/pmirGLO-MUT-CDK4, 7) miR-NC+pmirGLO-WT-lncRNA AFAP1-AS1-2/pmirGLO-WT-CDK4, or 8) miR-NC+pmirGLO-MUT-lncRNA AFAP1-AS1-2/pmirGLO-MUT-CDK4. Luciferase activity of MDA-MB-231 and BT-549 cells under each treatment was tested as per instructions of Dual-Luciferase Reporter Assay System kit (Promega, USA), which were repeated for ≥ 3 times.

### Statistical analyses

Data analyses in this investigation were fulfilled using SPSS ver.20 software (SPSS Inc. Chicago, IL, USA). Among them, quantitative data [mean ± standard deviation (SD)] were processed by student’s t-test or analysis of variance (ANOVA), and categorical data (n) were analyzed via chi-square test. Differences were statistically significant when two-sided *P* value was less than 0.05.

### Ethics approval and consent to participate

This investigation has obtained approvals from Longhua Hospital Affiliated to Shanghai University of TCM and the ethics committee of Longhua Hospital Affiliated to Shanghai University of TCM in advance.

### Availability of data and materials

The data used to support findings of this study are available from the corresponding author upon reasonable requests.

## Supplementary Material

Supplementary Figures

Supplementary Table 1

Supplementary Tables 2, 3, 4 and 5
