# Supplementary material for: Formononetin relieves the facilitating effect of lncRNA AFAP1-AS1-miR-195/miR-545 axis on progression and chemo-resistance of triple-negative breast cancer
Source: Aging (Albany NY). 2021 Jul 21;13(14):18191–222. doi: 10.18632/aging.203156 (PMC8351708; doi:10.18632/aging.203156)
Supplement: Supplementary Figures [file aging-13-203156-s001.pdf]

SUPPLEMENTARY FIGURES

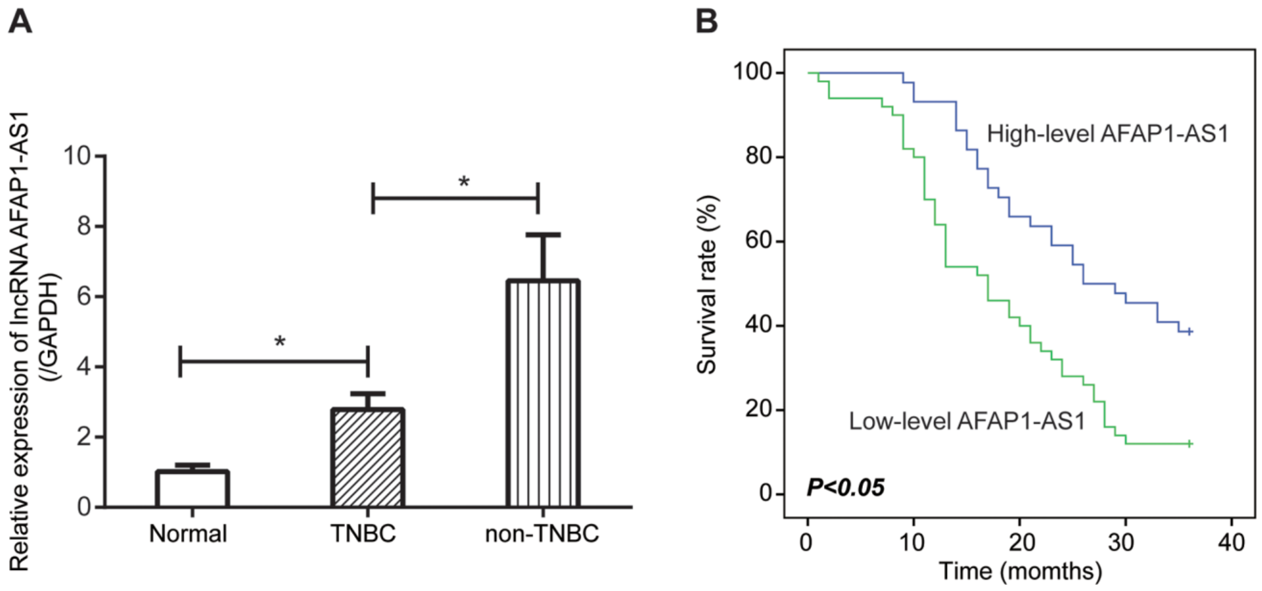

Supplementary Figure 1. Clinical significance of lncRNA AFAP1-AS1 in triple-negative breast cancer (TNBC). (**A**) LncRNA AFAP-AS1 expression was compared among adjacent normal tissues, TNBC tissues and non-TNBC tissues. *: *P*<0.05. (**B**) TNBC patients carrying low-level lncRNA AFAP-AS1 were more likely to enjoy favorable prognosis than patients with high lncRNA AFAP1-AS1 level.

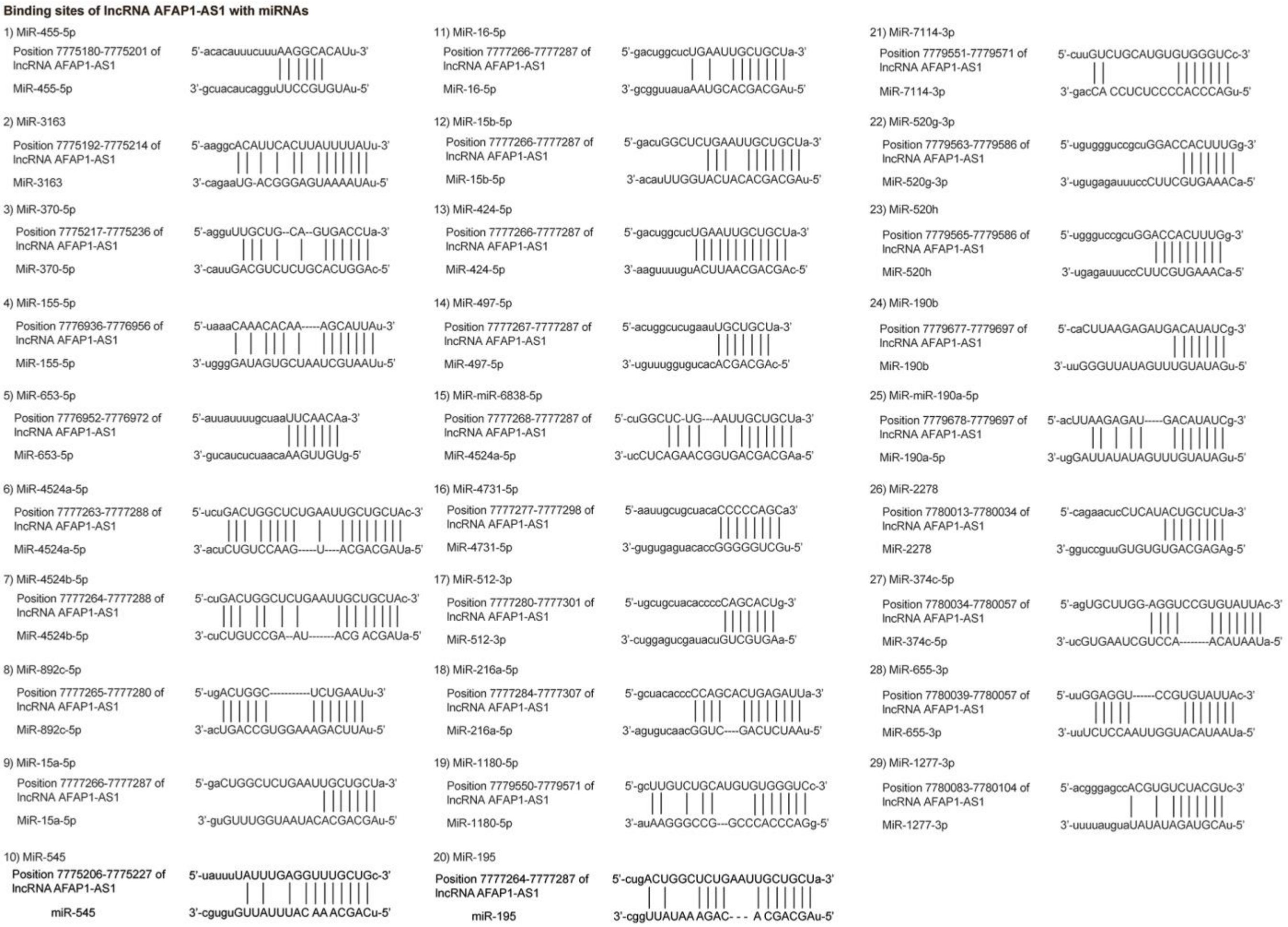

Supplementary Figure 2. Potential sponging sites between lncRNA AFAP1-AS1 and miRNAs in accordance with the Encyclopedia of RNA Interactomes (ENCORI) online database (http://starbase.sysu.edu.cn/).

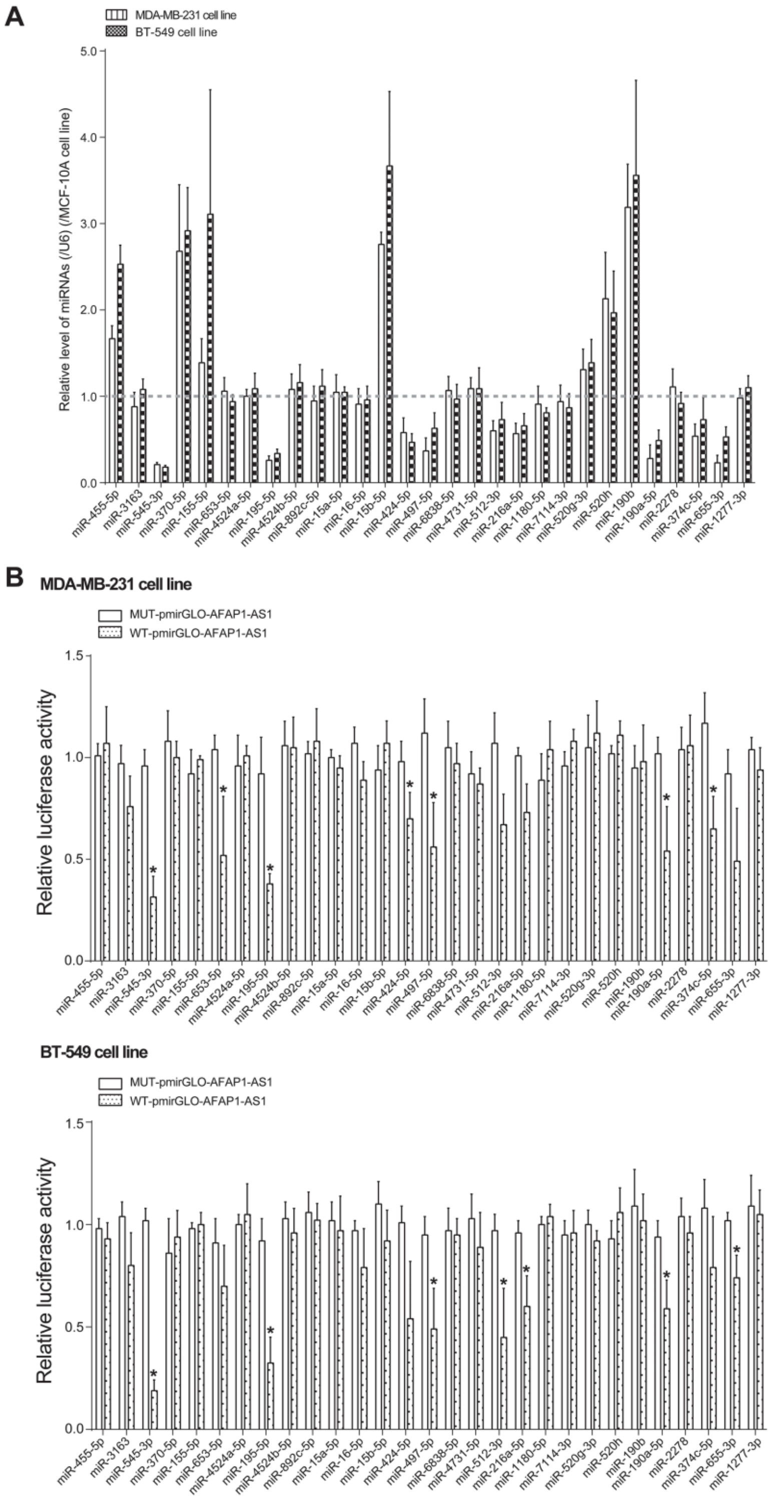

Supplementary Figure 3. MiRNAs potentially sponged by lncRNA AFAP1-AS1 were determined in MCF-10A, MDA-MB-231 and BT-549 cell lines (**A**), and luciferase activity of MDA-MB-231 and BT-549 cell lines were compared between pmirGLO-WT-AFAP1-AS1+pcDNA6.2/GW/EmGFP-miRNA group and pmirGLO-MUT-AFAP1-AS1+pcDNA6.2/GW/EmGFP-miRNA group (**B**). *: *P*<0.05 in comparison to pmirGLO-MUT-lncRNA AFAP1-AS1+pcDNA6.2/GW/EmGFP-miRNA group. Note: LncRNA AFAPA-AS1 fragments that contained binding sites of each miRNA were conserved and mutated, respectively, to construct WT-lncRNA AFAP1-AS1 and MUT-lncRNA AFAP1-AS1-1 for each miRNA. For each miRNA, the luciferase activity of MDA-MB-231/BT-549 cell line was compared between pmirGLO-WT-lncRNA AFAP1-AS1+pcDNA6.2/GW/EmGFP-miRNA group and pmirGLO-MUT-lncRNA AFAP1-AS1+pcDNA6.2/GW/EmGFP-miRNA group, both of which have been normalized to pmirGLO-WT-lncRNA AFAP1-AS1+pcDNA6.2/GW/EmGFP group.

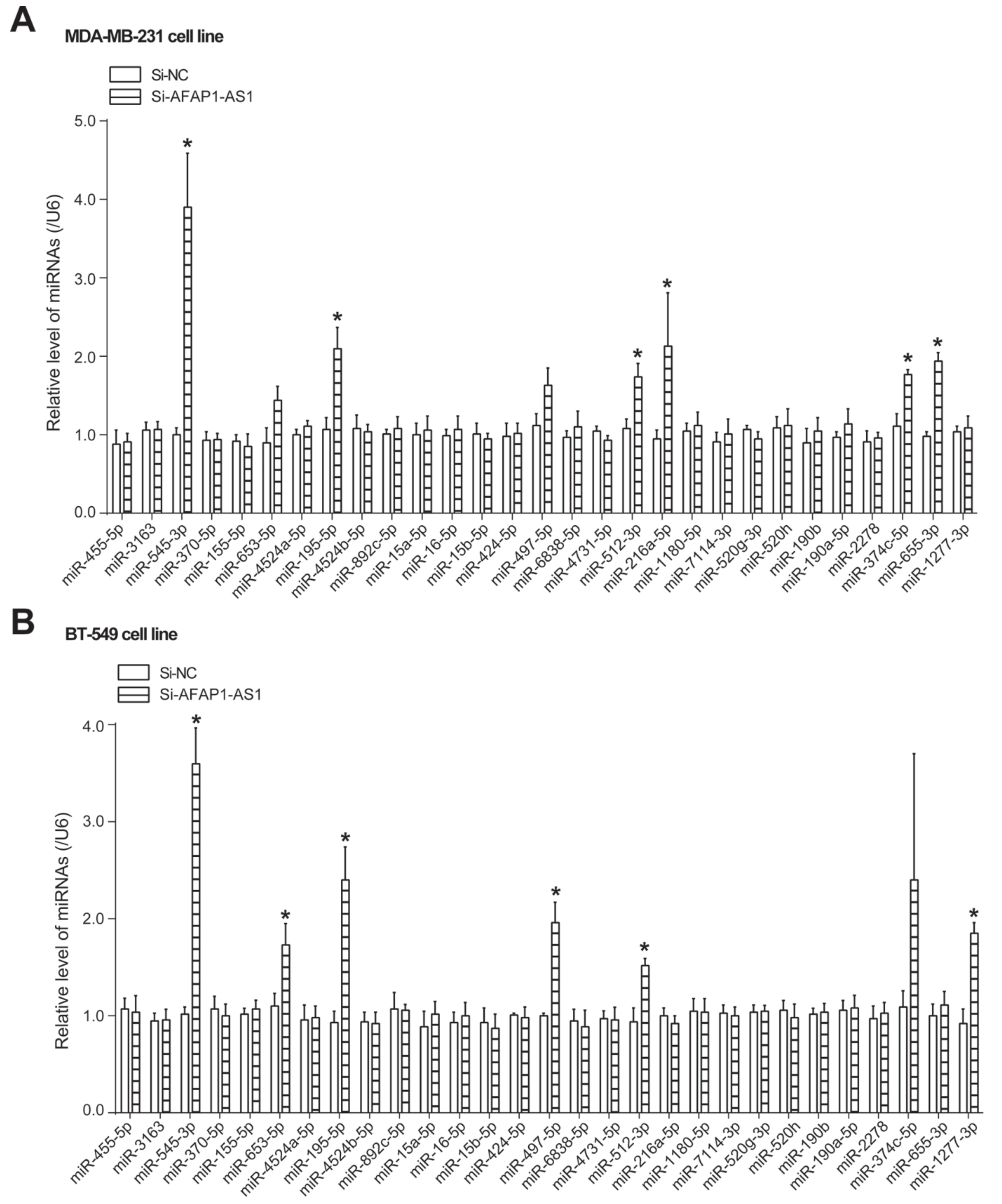

Supplementary Figure 4. MiRNAs potentially sponged by lncRNA AFAP1-AS1 were monitored in MDA-MB-231 (**A**) and BT-549 (**B**) cell lines after silencing of lncRNA AFAP1-AS1. *: *P*<0.05 in comparison to si-negative control (NC) group.

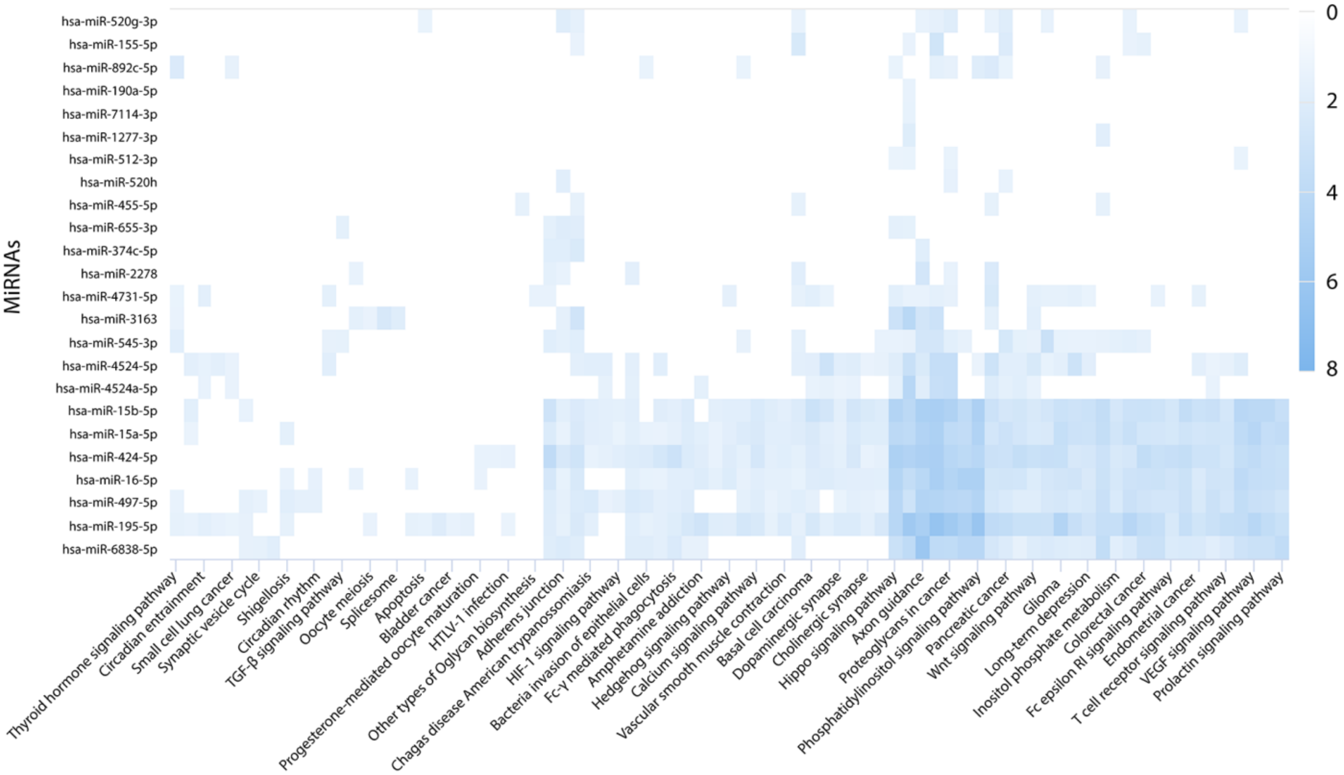

Supplementary Figure 5. KEGG pathways enriched by genes targeted by lncRNA AFAP1-AS1-sponged miRNAs, in the light of miRPathDB online database (https://mpd.bioinf.uni-sb.de/overview.html).

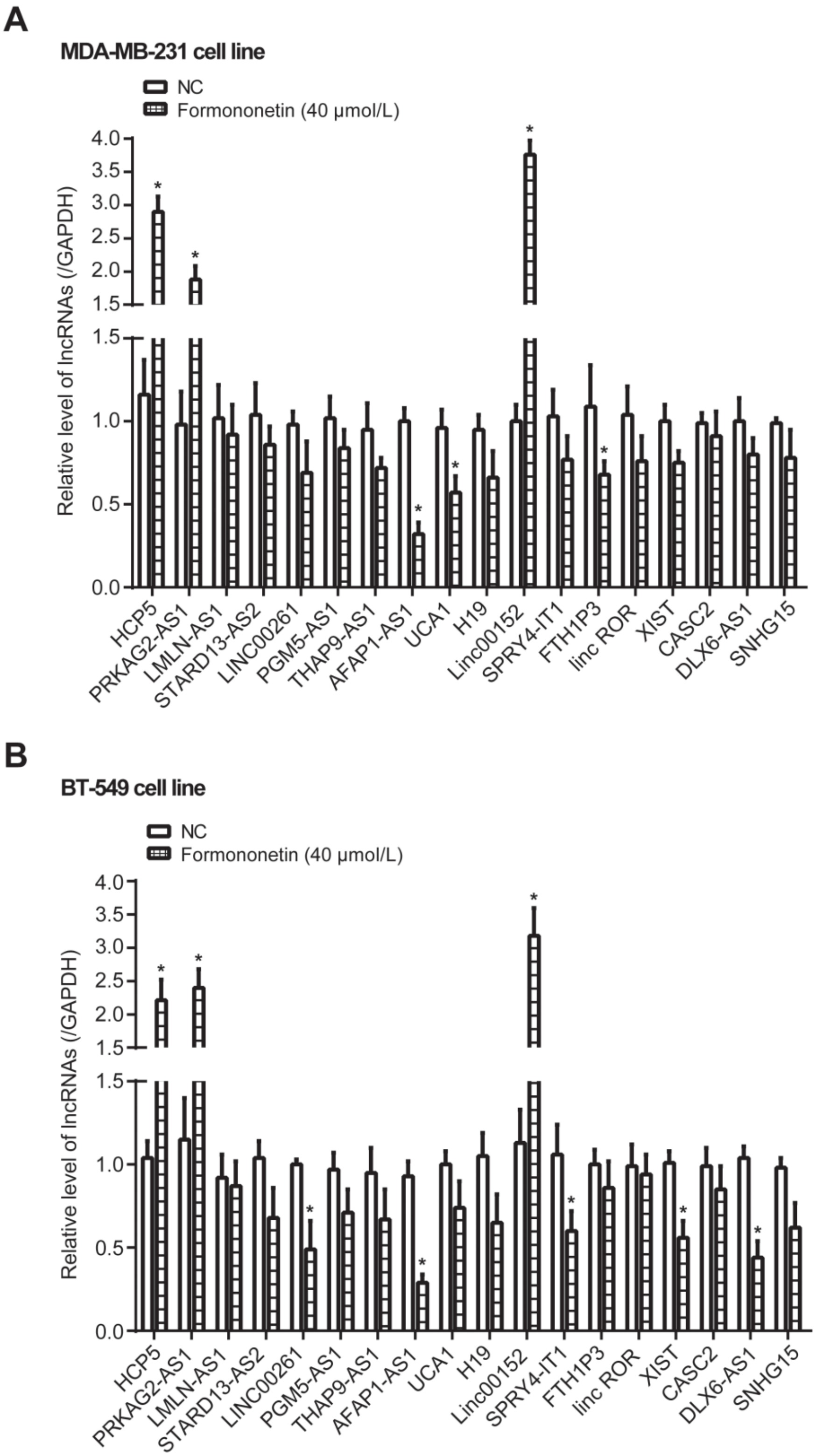

Supplementary Figure 6. Expressions of lncRNAs were measured in 40 μmol/L formononetin-exposed MDA-MB-231 (**A**) and BT-549 (**B**) cell lines. *: P<0.05 in comparison to negative control (NC) group.

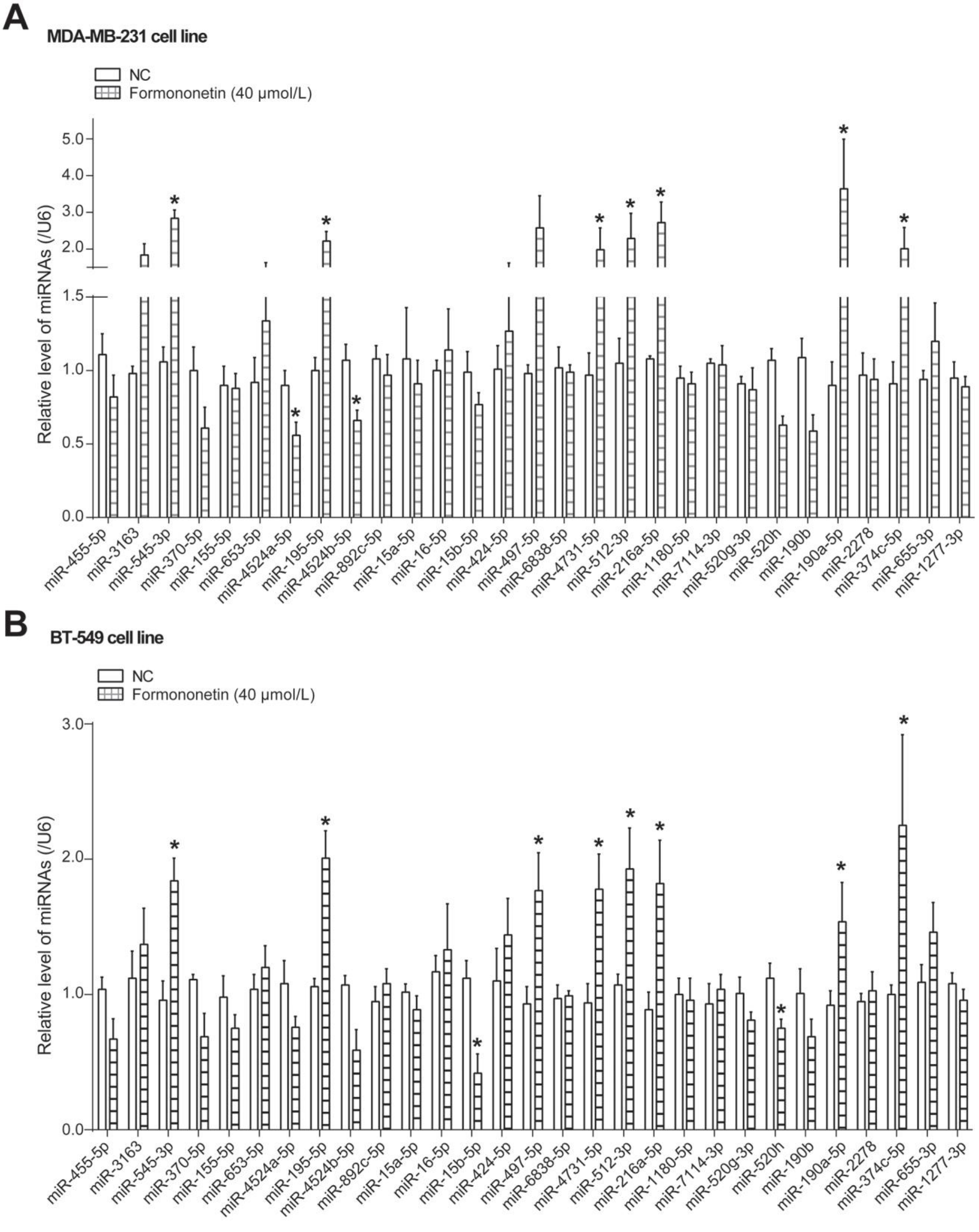

Supplementary Figure 7. Expressions of miRNAs were detected in MDA-MB-231 (**A**) and BT-549 (**B**) cell lines under treatment of 40 μmol/L formononetin. *: *P*<0.05 in comparison to negative control (NC) group.

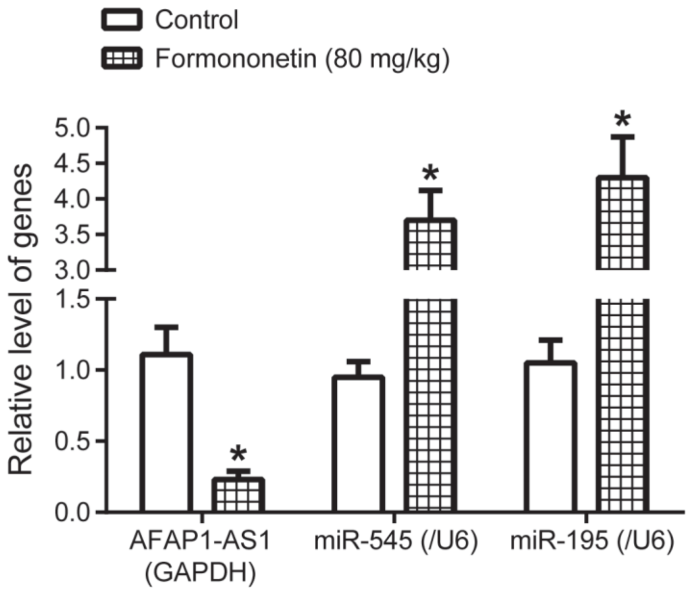

Supplementary Figure 8. LncRNA AFAP1-AS1, miR-545 and miR-195 expressions were determined in triple-negative breast cancer (TNBC)-bearing mice models after injection of 80 mg/kg formononetin. *: *P*<0.05 in comparison to control group.
