# Supplementary material for: Formononetin relieves the facilitating effect of lncRNA AFAP1-AS1-miR-195/miR-545 axis on progression and chemo-resistance of triple-negative breast cancer
Source: Aging (Albany NY). 2021 Jul 21;13(14):18191–222. doi: 10.18632/aging.203156 (PMC8351708; doi:10.18632/aging.203156)
Supplement: Supplementary Tables 2, 3, 4 and 5 [file aging-13-203156-s003.pdf]

SUPPLEMENTARY TABLES

Supplementary Table 2. Association of lncRNA AFAP1-AS1 expression with clinicopathological characteristics of breast cancer (BC) patients#.

Items	TNBC population (n=94)					Non-TNBC population (n=155)				
	High (n=51)	Low (n=43)	χ^2	*P*	OR (95%CI)	High (n=78)	Low (n=77)	χ^2	*P*	OR (95%CI)
**Age (years old, n)**										
≤55	33	34				55	45			
>55	17	10	1.453	0.228	1.752(0.701-4.38)	23	32	2.466	0.116	0.588(0.302-1.144)
**Histological grade (n)**										
I+II	15	26				45	40			
III	35	18	8.054	**0.005***	**3.37(1.436-7.908)**	33	37	0.516	0.472	0.793(0.421-1.494)
**Tumor size (n)**										
T1+T2	26	32				51	55			
T3	24	12	4.255	**0.039***	**2.462(1.036-5.847)**	27	22	0.655	0.418	1.324(0.671-2.612)
**Clinical stage (n)**										
I+II	33	27				58	57			
III	17	17	0.218	0.641	0.818(0.352-1.901)	20	20	0.002	0.962	0.983(0.479-2.018)
**Lymph-node metastasis (n)**										
No	19	27				35	38			
Yes	31	17	5.112	**0.024***	**2.591(1.126-5.963)**	43	39	0.312	0.577	1.197(0.637-2.251)
**Menopausal status (n)**										
No	27	22				41	33			
Yes	23	22	0.150	0.699	0.852(0.378-1.918)	37	44	1.463	0.226	0.677(0.359-1.275)
**Pathological type (n)**										
Invasive ductal carcinoma	43	40				61	59			
Others	7	4	0.546	0.460	1.628(0.443-5.986)	17	18	0.055	0.814	0.914(0.43-1.941)
**Family history (n)**										
No	39	29				54	59			
Yes	11	15	1.710	0.191	0.545(0.219-1.361)	24	18	1.072	0.301	1.457(0.713-2.975)
**Ki-67 (n)**										
≤14%	23	30				36	40			
>14%	27	14	4.683	**0.031***	**2.516(1.082-5.849)**	42	37	0.521	0.471	1.261(0.671-2.371)

#Collection of triple negative breast cancer (TNBC) specimens: Tumor tissues and adjacent normal tissues were collected from a total of 249 BC patients, who has been histopathologically confirmed as TNBC (n=94) and non-TNBC (n=155), recruited from Breast Department of Longhua Hospital Affiliated to Shanghai University of Traditional Chinese Medicine, from December of 2011 to November of 2016Participants in this program all met following criteria: 1) they were females with primary BC; 2) they underwent surgeries; 3) they have signed informed consents; and 4) their tissues were frozen within < 30 min since being excised. Moreover, the applicants were excluded if: 1) their TNBC subtype could not be verified; 2) their tissues were unavailable from surgery; 3) they participated in this program without consents from their family; and 4) their cancer tissues were not stored timely. This program was approved by Longhua Hospital Affiliated to Shanghai University of Traditional Chinese Medicine and the ethics committee of Longhua Hospital Affiliated to Shanghai University of Traditional Chinese Medicine. High: highly-expressed lncRNA AFAP1-AS1; Low: lowly-expressed lncRNA AFAP1-AS1; OR: odds ratio; CI: confidence interval; *: statistical significance.

Supplementary Table 3. Association of clinical parameters with prognosis of triple-negative breast cancer (TNBC) patients#.

Items	Number of cases (n)	Uni-variate analysis			Multi-variate analysis		
		HR	95% CI	*P* value	HR	95% CI	*P* value
**Age (years old, n)**							
≤55	67						
>55	27	0.919	0.547-1.5420.748	0.719	0.861	0.467-1.589	0.632
**Histological grade (n)**							
I+II	41						
III	53	**2.235**	**1.365-3.661**	0.001	1.566	0.935-2.623	0.088
**Tumor size (n)**							
T1+T2	58						
T3	36	**1.641**	**1.002-2.635**	0.040	**1.785**	**1.063-2.996**	**0.028***
**Clinical stage (n)**							
I+II	60						
III	34	**2.260**	**1.396-3.658**	0.001	**2.985**	**1.772-5.028**	**<0.001***
**Lymph-node metastasis (n)**							
No	46						
Yes	48	**2.080**	**1.298-3.335**	0.002	**2.354**	**1.408-3.933**	**0.001***
**Menopausal status (n)**							
No	49						
Yes	45	1.055	0.662-1.68	0.823	0.844	0.517-1.38	0.500
**Pathological type (n)**							
Invasive ductal carcinoma	83						
Others	11	0.656	0.3-1.433	0.290	1.095	0.47-2.552	0.834
**Family history (n)**							
No	68						
Yes	26	1.243	0.751-2.056	0.397	1.593	0.917-2.767	0.098
**Ki-67**							
≤14%	53						
>14%	41	**1.657**	**1.037-2.646**	0.035	1.521	0.865-2.674	0.146
**Relative expression of lncRNA AFAP1-AS1**							
Low expression	44						
High expression	50	**2.346**	**1.444-3.811**	0.001	**2.600**	**1.526-4.431**	**<0.001***

#Follow-up care: The TNBC patients were followed up from the date of diagnosis until December 31 of 2019. Their prognostic condition was tracked via telephone communication, and their clinical symptoms were re-examined during follow-up period. Parameters that affected prognosis of TNBC patients were figured out by establishing univariate and multivariate Cox regression models. HR: hazard ratio; CI: confidence interval; *: statistical significance.

Supplementary Table 4. Impact of formononetin on tumor growth in mice models#.

Group	Dose (mg/kg)	Body weight (g)		Tumor weight (g)	Inhibitory rate (%)
		Pre-treatment	Post- treatment		
Control	--	19.96±1.57	26.35±2.98	1.48±0.44	--
Formononetin	20	20.5±1.48	25.63±4.09	1.17±0.31	20.95%
	40	20.24±1.95	25.17±3.2	0.85±0.24	42.56%
	80	20.58±1.8	24.55±2.91	0.60±0.27*	66.22%

#: Establishment of triple negative breast cancer (TNBC)-bearing nude mice models: A total of 40 SPF-grade BALB/c female mice, aged 6.5 weeks, were provided by experimental animal center of Longhua Hospital Affiliated to Shanghai University of Traditional Chinese Medicine. After the mice were acclimate to surroundings at 25° C for 1week, they were injected by 0.1 ml MDA-MB-231 cell suspension (around 1 × 10^6 cells) subcutaneously in the chest. On the 2nd day, there existed a node in the injection site of each mouse, and TNBC-bearing mice models were established successfully. Then the mice models were divided into control group (n=10), formononetin (20 mg/kg) group (n=10), formononetin (40 mg/kg) group (n=10) and formononetin (80 mg/kg) group (n=10). Formononetin was intra-peritoneally injected into each mouse model once per day, for continuously 21 days. Body weight and tumor weight (W) of the mice models were recorded, and tumor growth inhibition rate (%), which was equivalent to $(1-W_{formononetin}/W_{control}) \times 100\%$, was calculated. What’s more, tumors of the mice models were excised to determine levels of lncRNA AFAP1-AS1-1, miR-545 and miR-195 with PCR. *: $P<0.05$ when compared with control group.

Supplementary Table 5. Primers of genes.

Genes	Primers	
	**Forward**	**Reverse**
LncRNAs		
HCP5	5'-ATGGTCCTGCTTTGGTGTCC-3'	5'-AGGCCCTACTTCTCTCAGGC-3'
PRKAG2-AS1	5'-CTGGAACCAGTAAGCCCGTT-3'	5'-GATCCACTGCGCAAACCTTG-3'
LMLN-AS1	5'-AGATTGCCTAGCAGAAGCCAG-3'	5'-TGGGTTTTGCTCTTGATTTAGCTC-3'
STARD13-AS2	5'-TTGGACCTCACCCAGGACTT-3'	5'-TGGGTATTTGCCTTGTGCCT-3'
LINC00261	5'-GCAATCCCCTCCTGAGCATT-3'	5'-CTCCACGGGCTACCAAATGT-3'
PGM5-AS1	5'-TGGTACTTTCAGCCTGTCCG-3'	5'-AACAGACGGCTTCAGTGGTT-3'
THAP9-AS1	5'-TCTTGGCATGGTTGGCTGTA-3'	5'-ATTCCTTCCCTGCATATTTTGAGT-3'
AFAP1-AS1	5'-GGAGTGACGGCATCCAACTC-3'	5'-GTCATCCCTGTCCCTGGTTC-3'
UCA1	5'-TGCCAGCCTCAGCTTAATCC-3'	5'-TCCCTGTTGCTAAGCCGATG-3'
H19	5'-CATGCTCCAGAGGGAATCGT-3'	5'-GCTTCAACTGATTCCGTGGC-3'
Linc00152	5'-CCAGCACCTCTACCTGTTGC-3'	5'-GCCAGACAAATGGGAAACCG-3'
SPRY4-IT1	5'-CCCAGAGAGCCAAGTCATCG-3'	5'-GGATGTTGGCATTCACAGGC-3'
FTH1P3	5'-CTCCTCCATTTACCTGTGCGT-3'	5'-CCGCACAGTCTGGTTTCTTG-3'
Linc ROR	5'-TCCTATGGAGGGGGAACCAT-3'	5'-GGAGTTCGACTTCCCCTGTG-3'
XIST	5'-GACACAAGGCCAACGACCTA-3'	5'-TCGCTTGGGTCCTCTATCCA-3'
CASC2	5'-TTGGTCTCGGGAACGTGAAG-3'	5'-CAACCAGGGAGGTGCTGAC-3'
DLX6-AS1	5'-GATATGGAACAGGCAAGCCG-3'	5'-ATGTTTGGAGGTTCCCCACC-3'
SNHG15	5'-TTGCCTGACCATTCCTGAGC-3'	5'-CCACTTTGAGACCGTCACCT-3'
*MiRNAs**		
MiR-455-5p	5'-GCGGCGGGCTACATCAGGTTTC-3'	5'-ATCCAGTGCAGGGTCCGAGG-3'
MiR-3163	5'-GCGGCGGCAGAATGACGGGAG-3'	5'-ATCCAGTGCAGGGTCCGAGG-3'
miR-545-3p	5'-GCGGCGGCGTGTGTTATTTAC-3'	5'-ATCCAGTGCAGGGTCCGAGG-3'
MiR-370-5p	5'-GCGGCGGCATTGACGTCTCTGC-3'	5'-ATCCAGTGCAGGGTCCGAGG-3'
MiR-155-5p	5'-GCGGCGGTGGGGATAGTGCTAATC-3'	5'-ATCCAGTGCAGGGTCCGAGG-3'
MiR-653-5p	5'-GCGGCGGGTCATCTCTAACAAAG-3'	5'-ATCCAGTGCAGGGTCCGAGG-3'
MiR-4524a-5p	5'-GCGGCGGACTCTGTCCAAGTAC-3'	5'-ATCCAGTGCAGGGTCCGAGG-3'
MiR-195-5p	5'-GCGGCGGCGGTTATAAAGACAC-3'	5'-ATCCAGTGCAGGGTCCGAGG-3'
MiR-4524b-5p	5'-GCGGCGGCTCTGTCCGAATACG-3'	5'-ATCCAGTGCAGGGTCCGAGG-3'
MiR-892c-5p	5'-GCGGCGGACTGACCGTGGAAAG-3'	5'-ATCCAGTGCAGGGTCCGAGG-3'
MiR-15a-5p	5'-GCGGCGGGTGTTTGGTAATACAC-3'	5'-ATCCAGTGCAGGGTCCGAGG-3'
MiR-16-5p	5'-GCGGCGGGCGGTTATAAATGCAC-3'	5'-ATCCAGTGCAGGGTCCGAGG-3'
MiR-15b-5p	5'-GCGGCGGACATTTGGTACTACAC-3'	5'-ATCCAGTGCAGGGTCCGAGG-3'
MiR-424-5p	5'-GCGGCGGAAGTTTTGTACTTAAC-3'	5'-ATCCAGTGCAGGGTCCGAGG-3'
MiR-497-5p	5'-GCGGCGGTGTTTGGTGTCACAC-3'	5'-ATCCAGTGCAGGGTCCGAGG-3'
MiR-6838-5p	5'-GCGGCGGTCCTCAGAACGGTGAC-3'	5'-ATCCAGTGCAGGGTCCGAGG-3'
MiR-4731-5p	5'-GCGGCGGGTGTGAGTACACCGG-3'	5'-ATCCAGTGCAGGGTCCGAGG-3'
MiR-512-3p	5'-GCGGCGGCTGGAGTCGATACTG-3'	5'-ATCCAGTGCAGGGTCCGAGG-3'
MiR-216a-5p	5'-GCGGCGGAGTGTCAACGGTCGAC-3'	5'-ATCCAGTGCAGGGTCCGAGG-3'
MiR-1180-5p	5'-GCGGCGGATAAGGGCCGGCCCAC-3'	5'-ATCCAGTGCAGGGTCCGAGG-3'
MiR-7114-3p	5'-GCGGCGGGACCACCTCTCCCCAC-3'	5'-ATCCAGTGCAGGGTCCGAGG-3'
MiR-520g-3p	5'-GCGGCGGTGTGAGATTTCCCTTC-3'	5'-ATCCAGTGCAGGGTCCGAGG-3'
MiR-520h	5'-GCGGCGGTGAGATTTCCCTTCG-3'	5'-ATCCAGTGCAGGGTCCGAGG-3'
MiR-190b	5'-GCGGCGGTTGGGTTATAGTTTG-3'	5'-ATCCAGTGCAGGGTCCGAGG-3'
MiR-190a-5p	5'-GCGGCGGTGGATTATATAGTTTG-3'	5'-ATCCAGTGCAGGGTCCGAGG-3'
MiR-2278	5'-GCGGCGGGGTCCGTTGTGTGTG-3'	5'-ATCCAGTGCAGGGTCCGAGG-3'

MiR-374c-5p	5'-GCGGCGGTCGTGAATCGTCCAAC-3'	5'-ATCCAGTGCAGGGTCCGAGG-3'
MiR-1277-3p	5'-GCGGCGGTTTTATGTATATATAG-3'	5'-ATCCAGTGCAGGGTCCGAGG-3'
MiR-655-3p	5'-GCGGCGGTTTCTCCAATTGGTAC-3'	5'-ATCCAGTGCAGGGTCCGAGG-3'
Others		
GAPDH	5'-GGAGCGAGATCCCTCCAAAAT-3'	5'-GGCTGTTGTCATACTTCTCATGG-3'
U6	5'-CGCTTACAGCAGACATAC-3'	5'-CGCTTACAGCAGACATAC-3'
Raf-1	5'-GGGAGCTTGGAAGACGATCAG-3'	5'-ACACGGATAGTGTTGCTTGTC-3'
CDK4	5'-ATGGCTACCTCTCGATATGAGC-3'	5'-CATTGGGGACTCTCACACTCT-3'

*: The miRNAs were potentially sponged by lncRNA AFAP1-AS1, as predicted by The Encyclopedia of RNA Interactomes (ENCORI) platform (http://starbase.sysu.edu.cn/).
